# Oxidized Hemithioindigo Photoswitches—Influence of Oxidation State on (Photo)physical and Photochemical Properties

**DOI:** 10.1002/chem.202002176

**Published:** 2020-07-23

**Authors:** Laura Köttner, Monika Schildhauer, Sandra Wiedbrauk, Peter Mayer, Henry Dube

**Affiliations:** ^1^ Department of Chemistry and Center for Integrated Protein Science CIPSM Ludwig-Maximilians-Universität München Butenandtstr. 5–13 81377 München; ^2^ Department of Chemistry and Pharmacy Friedrich-Alexander-Universität Erlangen-Nürnberg Nikolaus-Fiebiger-Str. 10 91058 Erlangen

**Keywords:** hemithioindigo, isomerization, photochemistry, photoswitches, physical chemistry

## Abstract

The photophysical and photochemical properties of sulfoxide and sulfone derivatives of hemithioindigo photoswitches are scrutinized and compared to the unoxidized parent chromophores. Oxidation results in significantly blue‐shifted absorptions and mostly reduction of photochromism while thermal stabilities of individual isomers remain largely unaltered. Effective photoswitching takes place at shorter wavelengths compared to parent hemithioindigos and high isomeric yields can be obtained reversibly in the respective photostationary states. Reversible solid‐state photoswitching is observed for a twisted sulfone derivative accompanied by visible color changes. These results establish oxidized hemithioindigo photoswitches as promising and versatile tools for robust light‐control of molecular behavior for a wide range of applications.

## Graphical abstract

**Light up your chemistry**! A series of sulfur‐oxidized hemithioindigo chromophores are introduced as versatile photoswitch motives for light‐controlled molecular behavior (see figure).

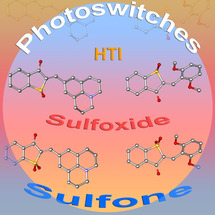

The hemithioindigo (HTI)^1^ structural motive belongs to the class of indigoid chromophores^2^ and has been known since 1906 when it was first synthesized by Paul Friedländer.^3^ It took some time since its discovery until HTI has been recognized to undergo light‐induced photoisomerization, which was first reported in 1961 by Mostoslavskii and co‐workers.^4^ Since then HTI has been applied as photoswitch by a couple of groups^5^ including our own,^6^ and has been further developed into a more mature photoswitching system primarily by the mechanistic works of Rück‐Braun, Cordes, Zinth,5f, 5h, 7 de Vivie‐Riedle,^8^ Riedle,^9^ and our own group.6a, 9, 10 Other research teams also have joined these efforts in recent years.^11^ In many studies the effects of various substitution patterns at different positions of the chromophore structure on the photo/physical properties have been explored quantitatively, which now enables conscious design of different property profiles of this class of photoswitches. At present it is thus well established that HTI chromophores possess many advantages permitting highly robust and efficient photoswitching within the visible part of the electromagnetic spectrum. When further exploring the structural motive of HTI, our group used oxidation of the sulfur atom to the corresponding sulfoxide as a straight‐forward method to introduce chirality into HTI chromophores with fourfold^12^ substituted central double bonds. Such structures enabled us to develop a variety of different molecular motor types that are responsive to visible light and perform unidirectional motions upon irradiation.^9^, ^13^ Despite this progress, the effects of sulfur oxidation on HTI photoswitches bearing three substituents at the central photoisomerizable double bond have to the best of our knowledge not been explored so far. Some photophysical data of mainly sulfone HTIs are described in the literature but no photoisomerization reactions are scrutinized.^14^ In this work we set out to fill in this gap and provide a survey of the effects of sulfur oxidation on the photoswitching properties of HTI. To this end we compare five parent HTI structures (**1**–**5**) with the corresponding sulfoxide (HTI‐SO **1**–**5**) and sulfone (HTI‐SO_2_ **1**–**5**) derivatives as shown in Figure 1 a. The specific substitution variations on the stilbene fragment were chosen because electronically neutral or donating substituents are generally beneficiary for HTI photoswitching.6a, 10a Likewise twisted structures such as in HTIs **4** and **5** have also been reported by our group to elicit interesting (photo)physical properties, especially with regard to high thermal bistability and switching performance.10c, 10f In this context it was deemed most effective to consistently increase the push‐pull character across the central double bond and evaluate the effects in a systematic way. For these reasons we increased acceptor strength of the thioindigo fragment by successive increase in the oxidation state of the sulfur atom and paired it with donor substituents on the stilbene fragment.

**Figure 1:**
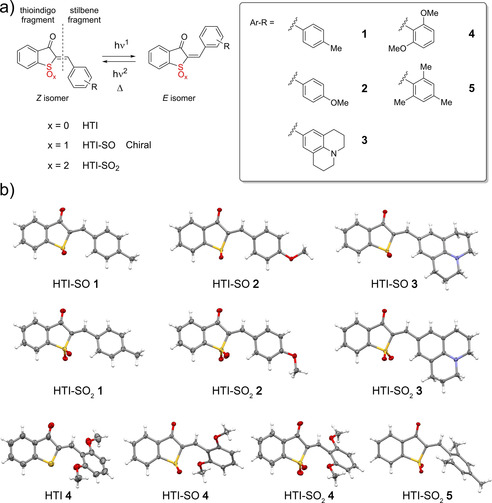
a) Molecular structures of parent HTIs **1**–**5** and oxidized derivatives HTI‐SO **1**–**5** and HTI‐SO_2_ **1**–**5**. b) Structures of HTI **4** and selected oxidized HTIs in their *Z* isomeric form in the crystalline state. HTI‐SO compounds **1**–**4** crystallized as racemic mixtures, only the (*S*)‐entiomeric forms are depicted for clarity.

Synthesis of oxidized HTI derivatives **1**–**5** followed either of two different approaches, oxidation of established HTI photoswitches using H_2_O_2_/AcOH or NaBO_3_ **⋅**4 H_2_O/AcOH, or condensation of oxidized benzothiophenones (sulfoxide **6** or sulfone **7**) with the corresponding aldehydes **8** or **9** (Scheme 1). Synthetic procedures are detailed in the Supporting Information together with comprehensive characterization (see also Figures S14–S25 for NMR spectra). Mixtures of the corresponding HTI‐SO and HTI‐SO_2_ products were usually obtained under the first conditions, which could easily be separated by conventional column chromatography. Typically the isolated yields of HTI‐SOs were much lower than of the corresponding HTI‐SO_2_s because of prolonged reaction times and an excess of H_2_O_2_ added (HTI‐SO : HTI‐SO_2_=5 % : 58 % for **2**, 26 % : 35 % for **4**). However, in the case of **5**, the opposite behavior was observed (HTI‐SO : HTI‐SO_2_=95 % : 5 %). HTI‐SO **1** was obtained after oxidation of the corresponding HTI **1** with NaBO_3_ **⋅**4 H_2_O in 20 % yield. HTI‐SO **3** was synthesized in 40 % yield by condensation of 9‐formyljulolidine (**8**) with known benzothiophenone‐sulfoxide (**6**).^15^ HTI‐SO_2_ derivatives **1** and **3** were obtained by condensation of the respective aldehydes **9** and **8** with commercially available benzothiophenone‐sulfone (**7**) in 50 % and 67 % yield, respectively. Crystals suitable for structural analysis were obtained for the *Z* isomers of HTI **4**, HTI‐SO **1** to **4**, and HTI‐SO_2_ derivatives **1**–**5** as shown in Figure 1 b (see also Tables S4–S8 in the Supporting Information). In the crystalline state it can be observed that the molecular structures of oxidized HTIs **1**–**3** are still almost completely planar structures facilitating efficient conjugation between the central photoisomerizable double bond and the stilbene fragments. This finding shows that oxidation at the sulfur atom does not induce substantial sterical hindrance at this position to lead to clashes with adjacent molecular fragments. However, the molecular structures of derivatives **4** and **5** are substantially distorted primarily because of the increased sterical hindrance induced by their methoxy and methyl substituents. The sterical effect is much more pronounced in the methyl‐substituted derivatives of HTI **5**.

**Scheme 1:**
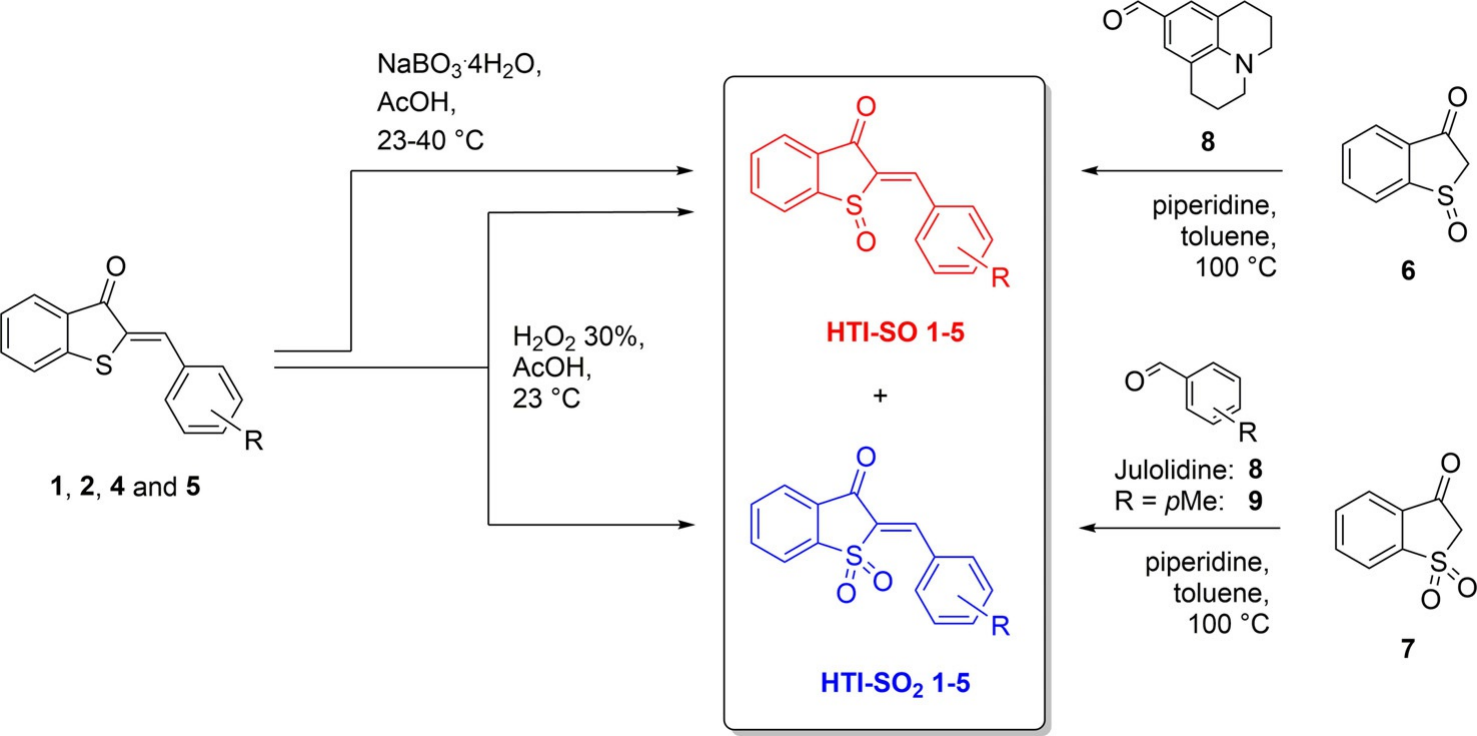
Different synthetic approaches to obtain oxidized HTI photoswitches **1**–**5**.

For many applications of photoswitches thermally stable switching states are required to ensure full light‐addressability of the system. Since metastable states are usually generated by at least one photoreaction, the kinetics of their spontaneous thermal decays are important parameters for photoswitches. For HTIs the *E* isomers are typically metastable and slowly decay thermally back to the *Z* isomers. We have measured the thermal decay of the *E* isomers for oxidized HTIs in toluene solution and analyzed them with the Eyring equation to derive the corresponding free activation enthalpies Δ*G* ^≠^ (for details see Figures S1–S3 and Tables S1 and S2 in the Supporting Information). All Δ*G* ^≠^ values are reported in Table 1. The free activation enthalpies for thermal double‐bond isomerizations range from 21.4 kcal mol^−1^ (HTI **3**) to >33.0 kcal mol^−1^ (HTI−SO and HTI−SO_2_ **5**), which is quite high compared to many conventional photoswitching systems. A possible reason for the significantly higher barriers of thermal isomerization of oxidized derivatives of HTI **5** may be rooted in a positive interaction between the oxygen atoms on the sulfur atom and the hydrogen atoms of the neighboring *ortho*‐methyl groups. Indications for this additional stabilization are derived from the relatively short distance between the oxygen atom and the corresponding methyl‐carbon atom in the crystal structures. When comparing the results with the unoxidized parent HTIs it can be seen that oxidation of the sulfur atom usually does not lead to significant effects with respect to the thermal isomerization reactions and no linear trends are observed for the changes seen. Free activation enthalpy differences Δ*G* ^≠^ in the range of 1.4 to 4.5 kcal mol^−1^ are observed upon oxidation of HTIs. These effects are not very strong for thermal double bond rotations rendering oxidized HTIs very bistable photoswitches as well. The only notable exception to this behavior is HTI system **4** for which oxidation leads to a significant reduction in thermal stability of up to 8.2 kcal mol^−1^ for HTI‐SO **4**. The resulting barrier of 23.3 kcal mol^−1^ is nevertheless still high enough to warrant bistable photoswitching for most applications.

**Table 1:** Quantitative comparison of the physical and photophysical properties of the structurally related HTIs, HTI‐SOs, and HTI‐SO_2_s **1**–**5**. Data were measured in CH_2_Cl_2_ or CD_2_Cl_2_ solutions.

HTI	*λ*_max_; *ϵ* of *Z* isomers most redshifted abs. [nm; L mol^−1^ cm^−1^]	*λ*_max_; *ϵ* of *E* isomers most redshifted abs. [nm; L mol^−1^ cm^−1^]	Isomer yield in the pss [%]/ [%] (nominal LED [nm])	Δ*G* ^≠^ (therm. *E*/*Z*)^[a]^ [kcal mol^−1^]	Half‐life of pure *E* isomer at 25 °C
**1**	435; 15 600	461; 9900	81 %/88 %^[a]^ *E* (420 nm) 100 %/95 %^[a]^ *Z* (490 nm)	30.9	164 a^[b]^
**SO‐1**	347; 21 200	350; 20 000	56 %/52 %^[a]^ *E* (305 nm) 73 %/73 %^[a]^ *Z* (385 nm)	29.7	22 a^[b]^
**SO_2_‐1**	353; 29 700	361; 24 000	62 %/64 %^[a]^ *E* (305 nm) 90 %/79 %* *Z* (405 nm)	28.2	1.7 a^[b]^
**2**	442; 17 900	467; 13 700	83 % *E* (420 nm) 100 % *Z* (530 nm)	26.4	3 d^[b]^
**SO‐2**	376; 29 400	385; 24 800	56 % *E* (365 nm) 93 % *Z* (450 nm)	30.9	164 a^[b]^
**SO_2_‐2**	383; 39 100	401; 31 700	70 % *E* (365 nm) 92 % *Z* (435 nm)	29.4	13 a^[b]^
**3**	500; 46 000	532; 40 000	82 %^[a]^ *E* (490 nm) 100 %^[a]^ *Z* (617 nm)	21.4	9 min
**SO‐3**	492; 42 700	496; 38 600	73 %^[a]^ *E* (470 nm) 100 %^[a]^ *Z* (530 nm)	22.8	1.6 h
**SO_2_‐3**	501; 39 200	502; 36 600	– –	–	–
**4**	442; 12 300	436; 6200	93 %/87 %^[a]^ *E* (365/420^[a]^ nm) 17 %/50 %^[a]^ *Z* (505/530^[a]^ nm)	31.7	633 a^[b]^
**SO‐4**	366; 15 400	356; 10 100	75 % *E* (365 nm) 76 % *Z* (435 nm)	23.3	3.8 h
**SO_2_‐4**	368; 18 300	365; 11 200	77 % *E* (365 nm) 83 % *Z* (435 nm)	24.7	1.7 d
**5**	420; 5900	425; 3600	85 % *E* (405 nm) 94 % *Z* (505 nm)	33.0	5700 a^[b]^
**SO‐5**	352; 5600	352; 6400	64 % *E* (365 nm) 95 % *Z* (435 nm)	>33.0	>5700 a^[b]^
**SO_2_‐5**	345; 3000	347; 7000 399; 3400	54 % *E* (365 nm) 95 % *Z* (435 nm)	>33.0	>5700 a^[b]^

[a] Measured in toluene or [D_8_]toluene solutions. [b] Linearly extrapolated approximations from high‐temperature measurements without taking into account temperature effects on Δ*G* ^≠^. The abbreviation “a” is used as SI‐unit for years.

For all chromophores reported here the molar absorptions were recorded in CH_2_Cl_2_ and/or toluene solution for both the *Z* and respective *E* isomers (see Figures S4–S6 in the Supporting Information). As it can clearly be seen in Figure 2 oxidation of the sulfur atom leads to a noticeable hypsochromic shift of the absorption. Interestingly, the first oxidation to the sulfoxide HTI‐SO derivatives induces the strongest hypsochromic shift of the absorption. Further oxidation to the sulfones HTI‐SO_2_ does not result in further hypsochromic shift. Instead the absorption remains essentially the same as compared to the corresponding sulfoxides. Apparently the electronic effects of sulfoxide or sulfone‐oxidation states are very similar in these chromophores. Hypsochromic shifting occurs for both isomers, but stronger so for the *E* isomers in most cases. The result is an overall reduction in the photochromism of these compounds. This observation can at least partly be explained by the more similar electron withdrawing character of the carbonyl and oxidized sulfur functions connected to the central double bond. Because of this similarity the electronic character of the molecule does not change very much when switching from the *Z* to the *E* isomer. The notable exception to this trend is the series of HTIs **4** where photochromism is actually improved slightly as shown in Figure 2 d. Upon oxidation of the sulfur atom molar absorption increases in comparison to the unoxidized parent HTIs in the series **1**, **2**, and **4** but decreases slightly for the series **3**. For series **5** the effects are more complicated with notable decreases of the *Z* isomer absorptions while the *E* isomers absorptions actually increase significantly upon oxidation. In all cases the typical two‐band structure of the absorption observed for unoxidized HTIs develops into a mainly single‐band structure for both HTI‐SO and HTI‐SO_2_ derivatives. With regard to solvent changes the absorptions of oxidized HTIs are not particularly solvatochromic except for derivatives bearing strong electron‐donor groups, that is, **3** (see Figure S7 and Table S3 in the Supporting Information). Absorption maxima and molar absorptions are summarized in Table 1 for all compounds.

**Figure 2:**
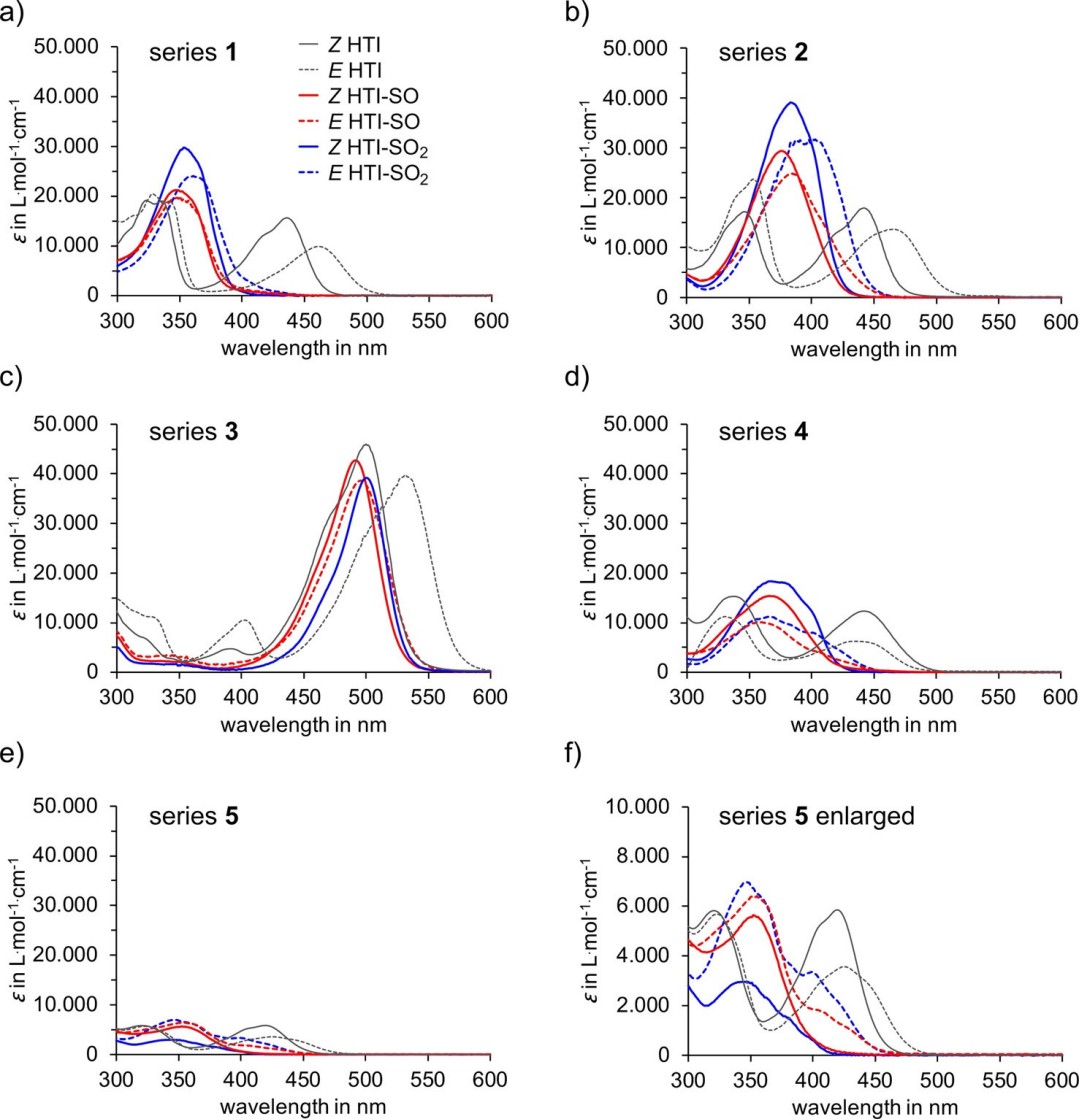
Comparison of molar absorptivities *ϵ* for the *Z* and *E* isomers of HTIs and the corresponding oxidized derivatives. Molar absorptivities were measured in CH_2_Cl_2_ solution for the series **2**, **4**, and **5** and in toluene solution for **1** and **3**. Spectra of HTIs are shown in grey, of sulfoxides HTI‐SOs in red, and of sulfones HTI‐SO_2_s in blue, of *Z* isomers as solid and of *E* isomers as broken lines (see legend in sub‐Figure a). a) Molar absorptivities of HTI series **1**. b) Molar absorptivities of HTI series **2**. c) Molar absorptivities of HTI series **3**. Note that molar absorptivities of the *E* isomer of **3**‐SO_2_ could not be obtained due to inefficient photoisomerization and strong signal overlapping of *Z* and *E* isomer in ^1^H NMR spectra. d) Molar absorptivities of HTI series **4**. e) Molar absorptivities of HTI series **5**. f) Enlarged section of the molar absorptivities of HTI series **5**.

Because of the less pronounced photochromism of oxidized HTI derivatives their photoisomerization reactions could be rendered less efficient in generating excess of a particular isomer. To test the photoswitching behavior, solutions with the respective oxidized HTI in either CH_2_Cl_2_ or toluene were irradiated to the photostationary state (pss) at different wavelengths and the maximum attainable isomer enrichment was quantified using ^1^H NMR spectroscopy (see Figures S8–S13 in the Supporting Information). The results are summarized in Table 1. As it turns out oxidized HTIs in general undergo quite efficient photoisomerizations with *E* isomer enrichment reaching up to 77 % and *Z* isomer enrichment reaching >90 % for the best performing derivatives as (shown exemplarily in Figure 3 for HTI‐SO **3** and **4** and HTI‐SO_2_ **2** and **4**). For all oxidized derivatives—except in the series of **3**—light with <400 nm is most efficient for the *Z* to *E* photoisomerization direction but also blue light of 405 nm is still effective in most cases. In general it can be seen that there is some decline in the achievable isomer enrichment in the photoisomerization reactions if the sulfur atom is oxidized. HTI‐SO derivatives are most of the time the least efficient photoswitches within a particular series and thus again no linear trend with increasing oxidation state is observed. In series **5** HTI‐SO is more efficient than HTI‐SO_2_ in the *Z* to *E* photoisomerization most likely because of the significantly higher absorption of the *E* isomer across the spectrum for the latter. The best performing sulfoxide photoswitches are HTI‐SO **3** and **4** reaching 73 % and 75 % *E* isomer enrichment as well as 100 % and 76 % *Z* isomer enrichment in the pss, respectively. The best performing sulfone photoswitches are HTI−SO_2_ **2** and **4** reaching 70 % and 77 % *E* isomer enrichment as well as 92 % and 83 % *Z* isomer enrichment in the pss, respectively. Oxidized HTI derivatives in the series **3** possessing strong electron donating groups lose photoswitching capability in more polar solvents, which is indicative of a possible competing deexcitation pathway that involves significant charge transfer in the excited state.10b, 10c For this reason photoisomerization reactions of derivatives **3** were measured in the less polar toluene solvent exclusively.

**Figure 3:**
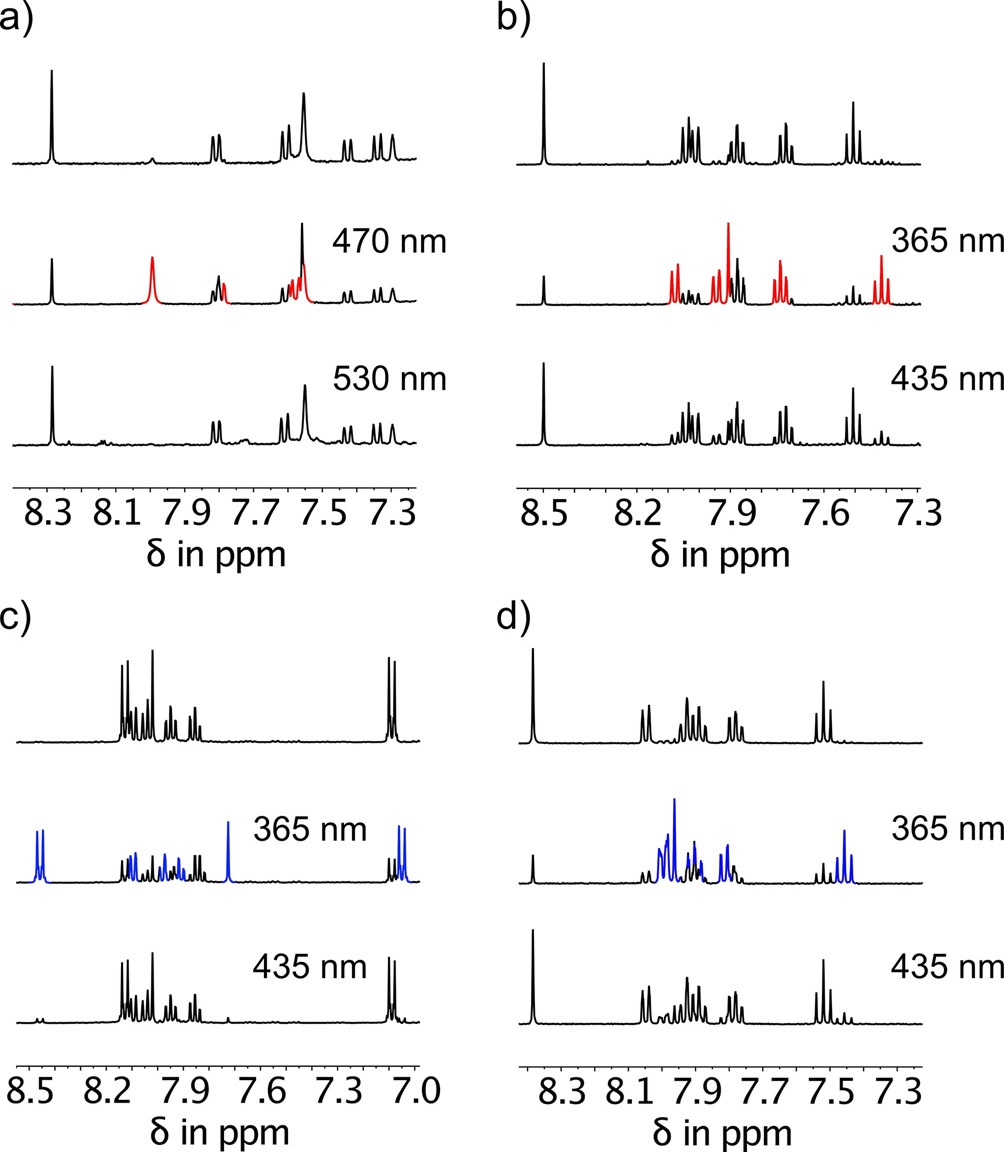
Photoswitching cycles of oxidized HTI derivatives in solution as followed by ^1^H NMR spectroscopy starting from pure *Z* isomers. Signals of photogenerated *E* isomers are highlighted in red for HTI‐SOs and in blue for HTI‐SO_2_s. a) Photoswitching of HTI‐SO **3** with blue (470 nm) and green (530 nm) light in [D_8_]toluene solution. b) Photoswitching of HTI‐SO **4** with UV (365 nm) and blue (435 nm) light in CD_2_Cl_2_ solution. c) Photoswitching of HTI‐SO_2_ **2** with UV (365 nm) and blue (435 nm) light in CD_2_Cl_2_ solution. d) Photoswitching of HTI−SO_2_ **4** with UV (365 nm) and blue (435 nm) light in CD_2_Cl_2_ solution.

It should be noted at this time that strong isomer enrichment is desirable for most photoswitching applications but is not necessary for applications in molecular machine building, especially molecular motors, where efficient photoswitching of both isomers is desirable at actually the same wavelength of irradiation. Therefore, it can be stated that oxidized HTIs possess high potential to serve as valuable molecular motives for both (visible)‐light induced photoswitching and molecular machine building.

As it was mentioned already above photochromism is reduced when oxidizing the sulfur atom and as a result changes in isomer composition are oftentimes not readily visible by the naked eye. The latter is also true for many unoxidized HTI photoswitches. This situation is different for the oxidized HTI series **1** where only the absorptions of the *E* isomers are crossing the 400 nm threshold. As a result colorless solutions of pure Z isomers turn yellow upon photoswitching for HTI‐SO **1** and HTI‐SO_2_ **1** (see Figure 4 also for a comparison of the photochromism within the full series **1**). A similar behavior is also observed for HTI‐SO_2_ **5**. However, for HTI‐SO_2_ **5** this easily visible color change is not confined to solution but also occurs in the solid and crystalline state evidencing effective photoswitching of this derivative in much more restricted environments. To illustrate this, we have irradiated amorphic films of the pure compound with alternating 365 nm and 420 nm light to reversibly affect color changes in the solid state (Figure 4 d). Such behavior is of great interest for optical materials applications for example, for information storage.

**Figure 4:**
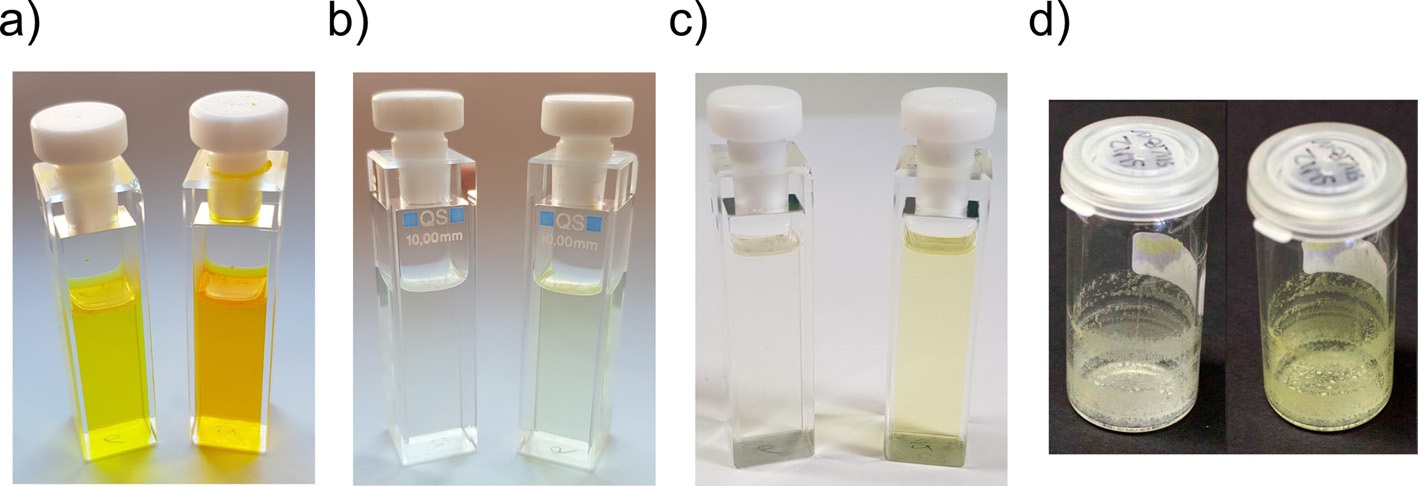
Color changes upon photoswitching of oxidized HTIs to the pss (*Z* isomers left, *E* isomer enriched right). a) Photoswitching of unoxidized HTI **1** in CH_2_Cl_2_ solution. b) Photoswitching of HTI‐SO **1** in CH_2_Cl_2_ solution. c) Photoswitching of HTI‐SO_2_ **1** in CH_2_Cl_2_ solution. d) Photoswitching of HTI‐SO_2_ **5** with 365 nm and 420 nm light in the solid state.

In summary we have compared the performance of a series of novel oxidized HTI photoswitches, both sulfoxide and sulfone derivatives, with their parent unoxidized HTI chromophores. We have shown that oxidized HTIs represent valuable novel photoswitches providing efficient photoisomerization reactions and high thermal stabilities of their metastable states. Particularly interesting are twisted derivatives that allow the observation of the photoisomerization process with the naked eye both in solution and solid state. Scrutiny of the chiroptical properties of chiral sulfoxide HTI‐SOs is currently underway in our laboratories and will be reported in due course. With these findings we hope to bring novel photoswitching motives to the attention of molecular scientists seeking reliable light responsive tools that elicit predictable structural changes upon irradiation.

## Conflict of interest

The authors declare no conflict of interest.

## Supporting information

As a service to our authors and readers, this journal provides supporting information supplied by the authors. Such materials are peer reviewed and may be re‐organized for online delivery, but are not copy‐edited or typeset. Technical support issues arising from supporting information (other than missing files) should be addressed to the authors.

Supplementary
