# Supplementary material for: Oxidized Hemithioindigo Photoswitches—Influence of Oxidation State on (Photo)physical and Photochemical Properties
Source: Chemistry. 2020 Jul 23;26(47):10712–8. doi: 10.1002/chem.202002176 (PMC7496871; doi:10.1002/chem.202002176)
Supplement: Supplementary file 1 — Supplementary [file CHEM-26-10712-s001.pdf]

Chemistry–A European Journal

Supporting Information

Oxidized Hemithioindigo Photoswitches—Influence of Oxidation State on (Photo)physical and Photochemical Properties

Laura Köttner+,[a] Monika Schildhauer+,[a] Sandra Wiedbrauk,[a] Peter Mayer,[a] and Henry Dube*[a, b]

Table of Contents

Materials and General Methods **2**

Synthesis of Compounds **4**

Thermal Isomerization **14**

Photophysical Properties **18**

Solvatochromic effects **21**

Photoswitching **22**

NMR Spectra **28**

Crystal Structural Data **40**

References **46**

Materials and General Methods

Reagents and solvents were obtained from *ABCR*, *Acros*, *Aldrich*, *Fluka, Merck, Sigma-Aldrich* or *TCI* in the qualities *puriss*., *p.a*., or *purum* and used as received. Technical solvents for extraction and column chromatography were distilled prior to use on a rotary evaporator (*Vacuubrand* CVC 3000). Reactions were monitored on *Merck* Silica 60 F254 TLC plates and detection was done by irradiation with UV light (254 nm or 366 nm).

Column chromatography was performed with silica gel 60 (*Merck*, particle size 0.063- 0.200 mm) and distilled technical solvents.

^{1}H NMR and ^{13}C NMR spectra were measured on a *Bruker AVANCE III HD 400* (400 MHz), *Varian VNMRS 40*0 (400 MHz), *Varian VNMRS 600* (600 MHz), or *Bruker AVANCE III HD 800* (800 MHz) NMR spectrometer. Deuterated solvents were obtained from *Cambridge Isotope Laboratories* and used without further purification. The chemical shifts are given in parts per million (ppm) on the delta scale (δ) relative to tetramethylsilane as external standard. Residual solvent signals in the ^{1}H and ^{13}C NMR spectra were used as internal reference. $CDCl_3$: δ_H = 7.26 ppm, δ_C = 77.2 ppm; CD_2Cl_2: δ_H = 5.32 ppm, δ_C = 54.0 ppm; toluene-d_8: δ_H = 2.09 ppm, δ_C = 20.4 ppm. The resonance multiplicity is indicated as *s* (singlet), *d* (doublet), *t* (triplet), *q* (quartet), *m* (multiplet). The coupling constant values (*J*) are given in hertz (Hz). Signal assignments are given in the experimental part with the arbitrary numbering indicated.

Electron Impact (EI) mass spectra were measured on a *Finnigan MAT95Q* or on a *Finnigan MAT90* mass spectrometer. The found masses from *high resolution* measurements (HR-EI-MS) are reported in *m*/*z* units with *M* as the molecular ion.

Melting points (m.p.) were determined on a *Büchi B-540 or EZ-Melt MPA 120* melting point apparatus in open capillaries.

Infrared spectra were measured on a *Perkin Elmer Spectrum BX-FT-IR* instrument equipped with a *Smith DuraSamplIR II* ATR-device. Transmittance values are qualitatively described by wavenumber (cm^{-1}) as strong (*s*), medium (*m*) and weak (*w*).

UV/vis spectra were recorded on a *Varian Cary 5000* spectrophotometer with a quartz cuvette (1 cm). Spectral grade solvents were obtained from *VWR* and *Merck*. Absorption wavelengths (λ) are given in nm and the molar absorption coefficients (ε) in $L \cdot mol^{-1} \cdot cm^{-1}$.

Photoisomerization experiments were conducted using LEDs from *Roithner Lasertechnik GmbH* (305 nm, 365 nm, 385 nm, 435 nm, 470 nm, 530 nm) for illumination. To specify the corresponding isomer

compositions in the PSS continuous irradiation of the solutions (CD_2Cl_2 and toluene-d_8) were carried out in NMR tubes at 23 °C. The ratios of the species were determined by ^{1}H NMR spectroscopy (400 MHz).

Synthesis of Compounds

Benzothiophenone **11**[1,2] and benzothiophenone-sulfoxide **6**[2,3] were synthesized based on established protocols. Benzothiophenone-sulfone **7** was obtained from *Aldrich*. 9-Formyljulolidin (**8**) was synthesized according to a published procedure.[1] Aldehydes **9** and **10** were obtained from *Sigma-Aldrich* or *TCI*. The general synthesis method of parent HTI **4** followed established protocols.[1,4] The synthesis of parent HTI **1** to parent HTI **3**[1] as well as parent HTI **5**[4] were also already reported.

Benzo[*b*]thiophen-3(*2H*)-one 1-oxide (6)[2,3]

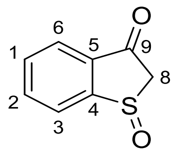

Benzo[*b*]thiopen-3(*2H*)-one (**11**) (0.63 g, 4.19 mmol, 1.0 eq.) was dissolved in conc. acetic acid (20 mL) and ethyl acetate (1 drop). $NaBO_3 \cdot 4\ H_2O$ (2.58 g, 16.76 mmol, 4.0 eq.) was added under N_2-athmosphere and stirred for 1.5 h at 23 °C. The reaction mixture was extracted with dichloromethane (3 x 150 mL), the organic phases were separated and dried over Na_2SO_4. After removing the solvent *in vacuo*, the crude product was purified by column chromatography (SiO_2, *i*Hex/EtOAc 95:5→1:1) and the product **6** was obtained as a pink solid (0.40 g, 2.42 mmol, 58%).

^{1}H NMR (600 MHz, CD_2Cl_2) δ (ppm) = 8.08 (*dt*, J = 7.8, 0.9 Hz, 1H, H-C(3)), 7.97 (*dt*, J = 7.7, 1.2, 0.7 Hz, 1H, H-C(6)), 7.93 (*ddd*, J = 7.8, 7.3, 1.2 Hz, 1H, H-C(2)), 7.78 (*td*, J = 7.5, 1.0 Hz, 1H, H-C(1)), 4.32 (*d*, J = 17.5 Hz, 1H, H_2-C(8)), 3.62 (*d*, J = 17.5 Hz, 1H, H_2-C(8)); ^{13}C NMR (101 MHz, CD_2Cl_2) δ (ppm) = 194.4 (C(9)), 154.4 (C(4)), 137.0 (C(2)), 133.7 (C(5)), 133.3 (C(1)), 128.2 (C(3)), 126.1 (C(6)), 61.2 (C(8)); HRMS (EI$^+$), $[M]^+$: *m/z* calcd. 166.0089 for $[C_8H_6O_2S]^+$, found 166.0083; R_f (SiO_2, *i*Hex/EtOAc 1:1) = 0.11.

(*Z*)-2-(2,6-Dimethoxybenzylidene)benzo[*b*]thiophen-3(2*H*)-one (HTI 4)

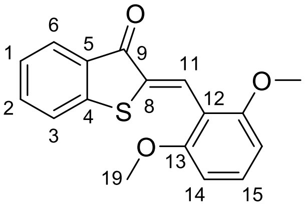

To a solution of 2-phenylthioacetic acid (3.20 g, 19.02 mmol, 4.8 eq.) in thionylchloride (12.3 mL, 168.62 mmol, 42.2 eq.) dimethylformamide (3 drops) was added. The reaction mixture was stirred for 12 h at 23 °C. Thionylchloride was removed *in vacuo* at 50 °C. The residual acid chloride was dissolved in 1,2-dichloroethane (50 mL) was added and the solution was cooled to 0 °C. $AlCl_3$ (3.10 g, 23.25 mmol, 5.8 eq.) was added in portions over a period of 2 min at 0 °C. The reaction mixture was stirred for 30 min at 0 °C and for 2 h at 23 °C. Ice/water (150 mL) was added and the aqueous phase was extracted with dichloromethane (3 · 50 mL). The organic phases were separated, dried over Na_2SO_4 and the organic solvent was removed *in vacuo.* The crude benzo[*b*]thiophen-3(2*H*)-one (**11**) was used for the next step without further purification. Benzo[*b*]thiophen-3(2*H*)-one (**11**) was dissolved in benzene (9.0 mL) and 2,6-dimethoxybenzaldehyde (**10**) (0.67 g, 4.00 mmol, 1.0 eq.) was added. The reaction mixture was stirred for 2 h at 100 °C. After cooling to 23 °C the reaction mixture was quenched with a saturated aqueous ammonium chloride solution (150 mL). The aqueous phase was extracted with ethyl acetate (3 x 200 mL) and the combined organic phases were dried over Na_2SO_4. After removing the solvent *in vacuo*, the crude product was purified by column chromatography (SiO_2, *i*Hex/EtOAc = 94:6) and the product HTI **4** was recrystallized from heptane to yield yellow crystals (0.51 g, 1.72 mmol, 43%).

^{1}H NMR (400 MHz, $CDCl_3$) δ (ppm) = 8.27 (*s*, 1H, H-C(11)), 7.88 (*ddd, J* = 7.7, 1.4, 0.7 Hz, 1H, H-C(6)), 7.51 (*ddd*, *J* = 7.9, 7.2, 1.4 Hz, 1H, H-C(2)), 7.41 (*ddd, J* = 7.9, 1.0, 0.7 Hz, 1H, H-C(3)), 7.41 (*t*, *J* =8.4 Hz, 1H, H-C(15)), 7.22 (*ddd*, *J* =7.7, 7.2, 1,0 Hz, 1H, H-C(1)), 6.59 (*d*, *J* = 8.4 Hz, 2H, H-C(14)), 3.91 (*s*, 6H, H_3-C(19)); ^{13}C NMR (101 MHz, $CDCl_3$) δ (ppm) = 189.1 (C(9)), 159.0 (C(13)), 147.0 (C(4)), 135.0 (C(2)), 133.4 (C(8)), 132.2 (C(15)), 131.3 (C(5)), 127.7 (C(11)), 126.8 (C(6)), 124.9 (C(1)), 123.4 (C(3)), 112.4 (C(12)), 103.7 (C(14)), 55.7 (C(19)); IR: $\tilde{\nu}$ (cm^{-1}) = 3323*w*, 2962*w*, 2931*w*, 2900*w*, 2832*w*, 1918*w*, 1804*w*, 1668*vs* 1592*vs*, 1562*vs*, 1469*s*, 1448*vs*, 1429*s*, 1353*w*, 1321*w*, 1310*m*, 1284*s*, 1276*s*, 1254*vs*, 1220*m*, 1200*s*, 1170*w*, 1158*w*, 1144*w*, 1112*vs*, 1092*m*, 1068*s*, 1055*vs*, 1028*s*, 1018*s*, 962*m*, 947*m*, 923*w*, 902*m*, 869*w*, 860*w*, 843*w*, 809*w*, 785*m*, 773*s*, 742*vs*, 728*vs*, 703*m*, 680*m*; M.p: 137 °C; HRMS (EI$^+$), [M]$^+$: *m/z* calcd. 298.0664 for [$C_{17}H_{14}O_3S$], found 298.0663; R_f(SiO_2, *i*Hex/EtOAc 8:2) = 0.37.

(*Z*)-2-(4-Methylbenzylidene)benzo[*b*]thiophen-3(2*H*)-one 1-oxide (HTI-SO 1)

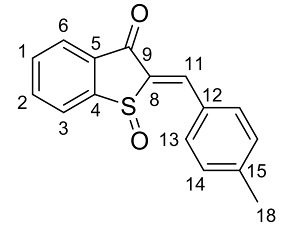

(Z)-2-(4-Methylbenzylidene)benzo[b]thiophen-3(2H)-one (HTI **1**) (150 mg, 0.59 mmol, 1.0 eq.) was dissolved in acetic acid (25 mL). $NaBO_3 \cdot 4\,H_2O$ (363 mg, 2.36 mmol, 4.0 eq.) was added and the reaction mixture was stirred for 19 h at 23 °C and for 4 h at 40 °C. Subsequently, a solution of NaOH solution (2 M, 300 mL) was added, the aqueous phase was extracted with dichloromethane (3 x 150 mL) and the combined organic phases were dried over Na_2SO_4. After removing the solvent *in vacuo*, the crude product was purified by column chromatography (SiO_2, *i*Hex/EtOAc = 95:5→1:1) and the product HTI-SO **1** was obtained as a yellow solid (32 mg, 0.12 mmol, 20%).

^{1}H NMR (600 MHz, CD_2Cl_2) δ (ppm) = 8.27 (*s*, 1H, H-C(11)), 8.12 (*dt*, J = 7.8, 0.8 Hz, 1H, H-C(3)), 8.07 (*dt*, J = 7.7, 0.9 Hz, 1H, H-C(6)), 8.01 (*d*, 2H, J = 8.12 Hz, H-C(13)), 7.93 (*td*, J = 7.5, 1.2 Hz, 1H, H-C(2)), 7.80 (*td*, J = 7.5, 1.0 Hz, 1H, H-C(1)), 7.40 (*dd*, J = 7.4, 1.2 Hz, 2H, H-C(14)), 2.46 (*s*, 3H, H_3-C(18)); ^{13}C NMR (150 MHz, $CDCl_3$) δ (ppm) = 185.9 (C(9)), 149.8 (C(4)), 146.8 (C(11)), 144.2 (C(15)), 137.2 (C(8)), 136.3 (C(2)), 133.4 (C(5)), 133.0 (C(1), C(13)), 130.2 (C(14)), 129.5 (C(12)), 127.5 (C(3)), 125.9 (C(6)), 21.5 (C(18)); IR: $\tilde{\nu}$ (cm^{-1}) = 2915*w*, 2208*w*, 2113*w*, 2011*w*, 1979*w*, 1951*w*, 1852*w*, 1716*w*, 1687*s*, 1595*s*, 1578*s*, 1560*s*, 1510*s*, 1449*m*, 1413*m*, 1345*m*, 1322*w*, 1311*w*, 1281*m*, 1227*w*, 1207*m*, 1187*m*, 1161*m*, 1115*m*, 1057*m*, 1015*s*, 989*m*, 974*m*, 957*w*, 916*w*, 876*w*, 816*m*, 783*m*, 746*s*, 707*m*, 669*m*, 655*m*; M.p.: 163 °C; HRMS (EI$^+$), [M]$^+$: *m/z* calcd. 268.0585 for $[C_{16}H_{12}O_2S]^+$, found 268.0567; R_f(SiO_2, *i*Hex/EtOAc 7:3) = 0.13.

(Z)-2-(4-Methoxybenzylidene)benzo[b]thiophen-3(2H)-one 1-oxide (HTI-SO 2)

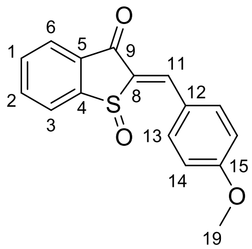

2-(4-Methoxybenzylidene)benzo[b]thiophen-3(2H)-one (HTI **2**) (50 mg, 0.19 mmol, 1.0 eq.) was dissolved in conc. acetic acid (1 mL). H_2O_2 (30%, 0.29 mL, 2.84 mmol, 14.9 eq.) was added and the reaction mixture was stirred for 25 h at 23 °C. Subsequently, the reaction was quenched with a saturated aqueous solution of $Na_2S_2O_3$ (10 mL), the aqueous phase was extracted with dichloromethane (3 x 50 mL). The combined organic phases were washed with a saturated aqueous solution of $NaHCO_3$ (50 mL) and dried over Na_2SO_4. After removing the solvent *in vacuo*, the crude product was purified by column chromatography (SiO_2, *i*Hex/EtOAc = 99:1→7:3, CD_2Cl_2/MeOH = 99:1) and the product HTI-SO **2** was obtained as a yellow solid (4 mg, 0.01 mmol, 5%).

^{1}H NMR (400 MHz, CD_2Cl_2) δ (ppm) = 8.24 (*s*, 1H, H-C(11))), 8.14 – 8.07 (*m*, 3H, H-C(3), H-C(13)), 8.05 (*dd*, *J* = 7.6, 1.3 Hz, 1H, H-C(6)), 7.92 (*td*, *J* = 7.5, 1.3 Hz, 1H, H-C(2)), 7.79 (*td*, *J* = 7.5, 1.0 Hz, 1H, H-C(1)), 7.09 (*d*, *J* = 8.86 Hz, 2H. H-C(14)), 3.92 (*s*, 3H, H_3-C(19)); ^{13}C NMR (150 MHz, $CDCl_3$) δ (ppm) = 185.8 (C(9)), 163.6 (C(15)), 149.5 (C(4)), 146.5 (C(11)), 136.1 (C(2)), 135.5 (C(13)), 135.3 (C(8)), 133.5 (C(5)), 132.9 (C(1)), 127.5 (C(3)), 125.8 (C(6)), 124.8 (C(12)), 115.0 (C(14)), 55.7 (C(19)); IR: $\tilde{\nu}$ (cm^{-1}) = 2842*w*, 2190*w*, 2136*w*, 2012*w*, 1993*w*, 1952*w*, 1675*s*, 1589*s*, 1558*s*, 1512*s*, 1451*m*, 1440*m*, 1427*m*, 1347*w*, 1334*w*, 1316*m*, 1301*m*, 1264*s*, 1186*s*, 1164*m*, 1115*m*, 1056*m*, 1029*s*, 1017*s*, 976*m*, 931*m*, 916*m*, 873*w*, 830*s*, 786*m*, 746*s*, 709*m*, 668*m*; M.p.: 171 °C; HRMS (EI$^+$), [M]$^+$: *m/z* calcd. 284.0507 for $[C_{16}H_{12}O_3S]^+$, found 284.0504; R_f (SiO_2, *i*Hex/EtOAc 1:1) = 0.28.

(*Z*)-2-((2,3,6,7-tetrahydro-1*H*,5*H*-pyrido[3,2,1-ij]quinolin-9-yl)methylene)benzo[*b*]thiophen-3(2*H*)-one 1-oxide (HTI-SO 3)

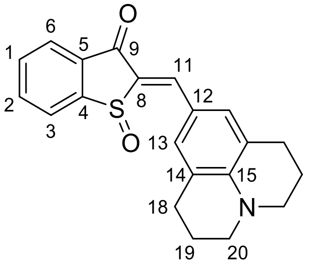

Benzo[*b*]thiophen-3(2*H*)-one 1-oxide (**6**) (121 mg, 0.60 mmol, 1.0 eq.) and 2,3,6,7-tetrahydro-1*H*,5*H*-pyrido[3,2,1-ij]quinoline-9-carbaldehyde (**8**) (116 mg, 0.70 mmol, 1.2 eq.) were dissolved in toluene (25 mL) and piperidine (2 drops) was added. After stirring for 5 h at 100 °C and cooling to 23 °C the reaction was quenched with a saturated aqueous solution of NH_4Cl (100 mL). The aqueous phase was extracted with ethyl acetate (3 x 100 mL) and the combined organic phases were dried over Na_2SO_4. After removing the solvent *in vacuo*, the crude product was purified by column chromatography (SiO_2, *i*Hex/EtOAc = 9:1→EtOAc) and the product HTI-SO **3** was obtained as a purple solid (84 mg, 0.24 mmol, 40%).

^{1}H NMR (400 MHz, CD_2Cl_2) δ (ppm) = 8.04 (*dt*, *J* = 7.5, 0.8 Hz, 1H, H-C(6)), 8.01 (*s*, 1H, H-C(11)), 7.97 (*ddd*, *J* = 7.6, 1.3, 0.7 Hz, 1H, H-C(3)), 7.82 (*td*, *J* = 7.5, 1.3 Hz, 1H, H-C(1)), 7.72 (*td*, *J* = 7.5, 1.0 Hz, 1H, H-C(2)), 7.56 (*s*, 2H, H-C(13)), 3.36 (*t*, 4H, H_2-C(20)), 2.81 (*t*, *J* = 6.3 Hz, 4H, H_2-C(18)), 2.02 – 1.94 (*m*, 4H, H_2- C(19)); ^{13}C NMR (101 MHz, $CDCl_3$) δ (ppm) = 185.8 (C(9)), 149.7 (C(5)), 148.5 (C(15)), 147.5 (C(11)), 135.5 (C(1)), 134.9 (C(4)), 134.4 (C(13)), 132.9 (C(2)), 129.6 (C(8)), 127.5 (C(6)), 125.7 (C(3)), 122.2 (C(14)), 119.0 (C(12)), 50.8 (C(20)), 28.3 (C(18)), 21.7 (C(19)); IR: $\tilde{\nu}$ (cm^{-1}) = 2952*w*, 2844*w*, 2519*w*, 2048*w*, 1978*w*, 1716*w*, 1664*w*, 1651*m*, 1614*w*, 1589*m*, 1552*m*, 1507*s*, 1464*m*, 1432*s*, 1417*s*, 1362*w*, 1349*w*,

1321*w*, 1258*s*, 1218*s*, 1206*m*, 1172*s*, 1162*s*, 1117*m*, 1082*s*, 987*m*, 956*m*, 918*m*, 900*m*, 876*m*, 788*m*, 758*w*, 748*s*, 732*m*, 701*m*, 684*s*, 654*m*; M.p.: 238 °C; HRMS (EI$^+$), [M]$^+$: *m/z* calcd. 349.1136 for $[C_{21}H_{19}NO_2S]^+$, found 349.1119; R_f(SiO_2, /EtOAc) = 0.30.

(*Z*)-2-(2,6-Dimethoxybenzylidene)benzo[*b*]thiophen-3(2*H*)-one 1-oxide (HTI-SO 4)

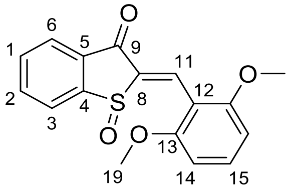

2-(2,6-Dimethoxybenzylidene)benzo[*b*]thiophen-3(2*H*)-one (HTI **4**) (60.0 mg, 0.20 mmol, 1.0 eq.) was dissolved in conc. acetic acid (2 mL) and H_2O_2 (30%, 0.30 mL, 2.94 mmol, 15.5 eq.) was added. After stirring for 17 h at 23 °C, the reaction mixture was quenched with a saturated aqueous solution of $Na_2S_2O_3$ (10 mL). The aqueous phase was extracted with dichloromethane (3 x 50 mL) and the combined organic phases were dried over Na_2SO_4. After removing the solvent *in vacuo*, the crude product was purified by column chromatography (SiO_2, *i*Hex/EtOAc = 99:1→7:3) and the product HTI-SO **4** was obtained as a yellow solid (17 mg, 0.05 mmol, 26%).

^{1}H NMR (600 MHz, CD_2Cl_2) δ (ppm) = 8.50 (*s*, 1H, H-C(11)), 8.04 (*d*, J = 7.5 Hz, 1H, H-C(6)), 8.01 (*d*, J = 7.8 Hz, 1H, H-C(3)), 7.88 (*t*, J = 7.5 Hz, 1H, H-C(1)), 7.72 (*t*, J = 7.5 Hz, 1H, H-C(2)), 7.51 (*t*, J = 8.4 Hz, 1H, H-C(15)), 6.69 (*d*, J = 8.4 Hz, 2H, H-C(14)), 4.01 (*s*, 6H, H_3-C(19)); ^{13}C NMR (101 MHz, CD_2Cl_2) δ (ppm) = 186.2 (C(9)), 160.5 (C(13)), 151.1 (C(5)), 141.5 (C(8)), 136.6 (C(11)), 136.6 (C(1)), 135.3 (C(15)), 134.9 (C(4)), 132.6 (C(2)), 127.4 (C(6)), 125.7 (C(3)), 110.5 (C(12)), 104.2 (C(14)), 56.3 (C(19)); IR: $\tilde{\nu}$ (cm^{-1}) = 2938*vw*, 2838*vw*, 1693*w*, 1592*m*, 1570*m*, 1472*m*, 1431*m*, 1329*w*, 1282*w*, 1255*m*, 1230*w*, 1202*w*, 1174*w*, 1113*s*, 1058*m*, 1030*m*, 1022*m*, 962*w*, 932*w*, 881*w*, 870*w*, 851*w*, 810*vw*, 786*w*, 776*m*, 751*s*, 729*m*, 681*m*, 667*w*; M.p.: 198 °C; HRMS (EI$^+$), [M]$^+$: *m/z* calcd. 314.0613 for $[C_{17}H_{14}O_4S]^+$, found. 314.0606; R_f(SiO_2, *i*Hex/EtOAc 1:1) = 0.07.

(*Z*)-2-(2,4,6-Trimethylbenzylidene)benzo[*b*]thiophen-3(2*H*)-one 1-oxide (HTI-SO 5)

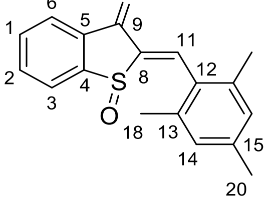

2-(2,4,6-Trimethylbenzylidene)benzo[*b*]thiophen-3(2*H*)-one (HTI **5**) (30 mg, 0.11 mmol, 1.0 eq.) was dissolved in conc. acetic acid (1 mL). H_2O_2 (30%, 0.15 mL, 1.47 mmol, 13.3 eq.) was added and the reaction mixture was stirred for 23 h at 23 °C. Subsequently, a saturated aqueous solution of $Na_2S_2O_3$ (10 mL) was added, the aqueous phase was extracted with dichloromethane (3 x 20 mL) and the combined organic phases were dried over Na_2SO_4. After removing the solvent *in vacuo*, the crude product was purified by column chromatography (SiO_2, *i*Hex/EtOAc = 99:1→7:3) and the product HTI-SO **5** was obtained as a yellow solid (33 mg, 0.11 mmol, 95%).

^{1}H NMR (400 MHz, CD_2Cl_2) δ (ppm) = 8.42 (*s*, 1H, H-C(11)), 8.09 (*d*, *J* = 7.7 Hz, 1H, H-C(3)), 8.04 (*d*, *J* = 7.7 Hz, 1H, H-H(6)), 7.92 (*td*, *J* = 7.5, 1.3 Hz, 1H, H-C(1)), 7.80 (*td*, *J* = 7.5, 1.1 Hz, 1H, H-C(2)), 6.98 (*s*, 2H, H-C(14)), 2.34 (*s*, 3H, H_3-C(20)), 2.25 (*s*, 6H, H_3-C(18)); ^{13}C NMR (101 MHz, CD_2Cl_2) *δ* (ppm) = 185.3 (C(9)), 151.1 (C5)), 148.0 (C(11)), 145.3 (C(8)), 139.9 (C(15)), 137.2 (C(1)), 136.1 (C(13)), 134.5 (C(4)), 133.6 (C(2)), 130.0 (C(12)), 129.0 (C(14)), 128.4 (C(6)), 126.5 (C(3)), 21.5 (C(20)), 20.9 (C(18)); IR: $\tilde{\nu}$ (cm^{-1}) = 2956*w*, 2920*m*, 2851*w*, 1693*s*, 1622*m*, 1579*m*, 1458*w*, 1449*m*, 1377*w*, 1324*vw*, 1277*m*, 1211*w*, 1162*w*, 1112*w*, 1062*s*, 1037*s*, 961*vw*, 943*vw*, 916*w*, 896*vw*, 864*m*, 798*vw*, 786*vw*, 772*vw*, 745*s*, 726*w*, 707*m*, 685*s*, 664*w*; M.p.: 185 °C; HRMS (EI^+), $[M]^+$: *m/z* calcd. 296.0871 for $[C_{18}H_{16}O_2S]^+$, found 296.0865; R_f(SiO_2, *i*Hex/EtOAc 7:3) = 0.16.

(*Z*)-2-(4-Methoxybenzylidene)benzo[*b*]thiophen-3(2*H*)-one 1,1-dioxide (HTI-SO_2 1)

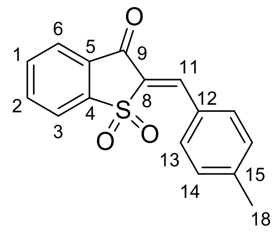

Benzo[*b*]thiophen-3(2*H*)-one 1,1-dioxide (**7**) (100 mg, 0.55 mmol, 1.0 eq.) and 4-methylbenzaldehyde (**9**) (66 mg, 0.55 mmol, 1.0 equiv.) were dissolved in toluene (20 mL) and piperidine (2 drops) was added. After stirring for 17 h at 23 °C and 1.5 h at 100 °C, the reaction mixture was cooled to 23 °C and quenched with a saturated aqueous solution of NH_4Cl (50 mL). The aqueous phase was extracted with ethyl acetate (3 x 50 mL) and the combined organic phases were dried over Na_2SO_4. After removing the solvent *in vacuo*, the crude product was purified by column chromatography (SiO_2, *i*Hex/EtOAc = 95:5→7:3) and the product HTI-SO_2 **1** was obtained as a yellow solid (78 mg, 0.27 mmol, 50%).

^{1}H NMR (600 MHz, CD_2Cl_2) *δ* (ppm) = 8.11 (*dt*, *J* = 7.7, 0.9 Hz, 1H, H-C(3)), 8.07 – 8.02 (*m*, 5H, H-(6), H-C(11), H-C(13)), 7.96 (*td*, *J* = 7.6, 1.2 Hz, 1H, H-C(1)), 7.86 (*td*, *J* = 7.5, 1.1 Hz, 1H, H-C(2)), 7.40 (*d*, *J* = 8.1 Hz, 2H, H-C(14)), 2.47 (*s*, 3H, H_3-C(18)); ^{13}C NMR (101 MHz, CD_2Cl_2) *δ* (ppm) = 178.8 (C(9)),

145.5 (C(15)), 144.9 (C(11)), 144.3 (C(4)), 136.6 (C(2)), 134.2 (C(1)), 133.5 (C(13), C8)), 132.4 (C(5)), 130.1 (C(14)), 127.9 (C(12)), 124.7 (C(6)), 121.2 (C(3)), 21.7 (C(18)); IR: $\tilde{\nu}$ (cm^{-1}) = 2998*w*, 2912*w*, 1809*w*, 1679*s*, 1591*m*, 1567*m*, 1557*m*, 1509*m*, 1449*m*, 1408*w*, 1377*w*, 1332*w*, 1281*s*, 1221*m*, 1206*m*, 1155*w*, 1129*w*, 1067*s*, 1056*s*, 1016*m*, 944*w*, 915*w*, 905*m*, 810*s*, 776*m*, 759*m*, 744*m*, 716*m*, 675*s*, 656*m*; M.p.: 184 °C; HRMS (EI^+), $[M]^+$: *m/z* calcd. 284.0507 for $[C_{16}H_{12}O_3S]^+$, found 284.0503; R_f(SiO_2, *i*Hex/EtOAc 9:1) = 0.14.

(*Z*)-2-(4-Methoxybenzylidene)benzo[*b*]thiophen-3(2*H*)-one 1,1-dioxide (HTI-SO_2 2)

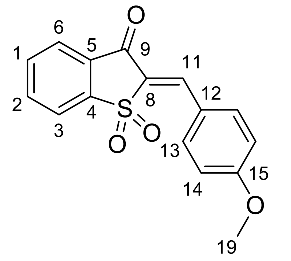

2-(4-Methoxybenzylidene)benzo[*b*]thiophen-3(2*H*)-one (HTI **2**) (50 mg, 0.19 mmol, 1.0 eq.) was dissolved in conc. acetic acid (1 mL). H_2O_2 (30%, 0.29 mL, 2.84 mmol, 14.9 eq.) was added and the reaction mixture was stirred for 25 h at 23 °C. Subsequently, a saturated aqueous solution of $Na_2S_2O_3$ (10 mL) was added, the aqueous phase was extracted with dichloromethane (3 x 50 mL), the combined organic phases were washed with a saturated aqueous solution of $NaHCO_3$ (50 mL) and dried over Na_2SO_4. After removing the solvent *in vacuo*, the crude product was purified by column chromatography (SiO_2, *i*Hex/EtOAc = 99:1→8:2) and the product HTI-SO_2 **2** was obtained as a yellow solid (33 mg, 0.11 mmol, 58%).

^{1}H NMR (600 MHz, CD_2Cl_2) δ (ppm) = 8.13 (*d*, *J* = 8.9 Hz, 1H, H-C(13)), 8.09 (*d*, *J* = 7.7 Hz, 1H, H-C(3)), 8.05 (*d*, *J* = 7.8 Hz, 1H, H-C(6)), 8.02 (*s*, 1H, H-C(11)), 7.95 (*td*, *J* = 7.6, 1.2 Hz, 1H, H-C(1)), 7.85 (*td*, *J* = 7.5, 1.1 Hz, 1H, H-C(2)), 7.12 – 7.06 (*m*, 2H, H-C(14)), 3.93 (*s*, 3H, H_3-C(19)); ^{13}C NMR (101 MHz, CD_2Cl_2) δ (ppm) = 179.3 (C(9)), 165.0 (C(15)), 145.1 (C(11)), 144.7 (C(5)), 137.0 (C(1)), 136.9 (C(13)), 134.7 (C(2)), 133.1 (C(4)), 128.7 (C(8)), 125.1 (C(3)), 123.9 (C(12)), 121.6 (C(6)), 115.5 (C(14)), 56.3 (C(19)); IR: $\tilde{\nu}$ (cm^{-1}) = 3112*w*, 2974*w*, 2934*w*, 2842*w*, 1731*w*, 1693*w*, 1588*w*, 1559*m*, 1512*m*, 1458*w*, 1441*w,* 1429*w*, 1350*w*, 1330*w*, 1320*w*, 1285*m*, 1270*s*, 1211*w*, 1184*s*, 1155*s*, 1119*m*, 1067*m*, 1046*m*, 1021*m*, 1010*m*, 966*w*, 942*m*, 924*m*, 836*s*, 782*m*, 761*w*, 744*s*; M.p.: 171 °C; HRMS (EI^+), $[M]^+$: *m/z* calcd. 300.0456 for $[C_{16}H_{12}O_4S]^+$, found 300.0450; R_f(SiO_2, *i*Hex/EtOAc 7:3) = 0.28.

(*Z*)-2-((2,3,6,7-Tetrahydro-1*H*,5*H*-pyrido[3,2,1-ij]quinolin-9-yl)methylene)benzo[*b*]thiophen-3(2*H*)-one 1,1-dioxide (HTI-SO_2 3)

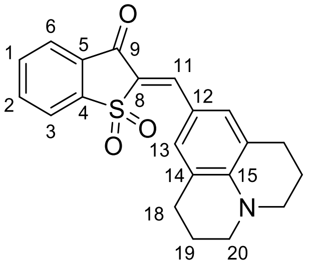

Benzo[*b*]thiophen-3(2*H*)-one 1,1-dioxide (**7**) (199 mg, 1.10 mmol, 1.0 eq.) and 2,3,6,7-tetrahydro-*1H,5H*-pyrido[3,2,1-ij]quinoline-9-carbaldehyde (**8**) (336 mg, 1.67 mmol, 1.5 eq.) were dissolved in toluene (30 mL) and piperidine (2 drops) was added. After stirring for 6 h at 100 °C and cooling to 23 °C, the reaction was quenched with a saturated aqueous solution of NH_4Cl (100 mL). The aqueous phase was extracted with ethyl acetate (3 x 150 mL) and the combined organic phases were dried over Na_2SO_4. After removing the solvent *in vacuo*, the crude product was purified by column chromatography (SiO_2, *i*Hex/EtOAc = 95:5→7:3) and the product HTI-SO_2 **3** was obtained as a yellow solid (268 mg, 0.73 mmol, 67%).

^{1}H NMR (600 MHz, CD_2Cl_2) δ (ppm) = 8.00 (*dd*, *J* = 7.6, 1.0 Hz, 1H, H-C(6)), 7.96 (*dd*, *J* = 7.7, 1.0 Hz, 1H, H-C(3)), 7.84 (*td*, *J* = 7.5, 1.1 Hz, 1H, H-C(2)), 7.79 – 7.75 (*m*, 2H, H-C(1), H-C(11)), 7.60 (*s*, 2H, H-C(13)), 3.39 (*t*, *J* = 5.8 Hz, 4H, H_2-C(20)), 2.79 (*t*, *J* = 6.3 Hz, 4H, H_2-C(18)), 1.98 (*p*, *J* = 6.1 Hz, 4H, H_2-C(19)); ^{13}C NMR (150 MHz, CD_2Cl_2) δ (ppm) = 178.8 (C(9)), 149.6 (C(15)), 145.0 (C(11)), 144.4 (C(4)), 135.7 (C(2)), 135.2 (C(13)), 134.1 (C(1)), 133.8 (C(5)), 124.5 (C(6)), 122.2 (C(14)), 122.0 (C(8)), 121.1 (C(3)), 117.9 (C(12)), 51.1 (C(20)), 28.2 (C(18)), 21.6 (C(19)); IR: $\tilde{\nu}$ (cm^{-1}) = 2914*w*, 2944*w*, 2844*w*, 1882*w*, 1724*w*, 1671*m*, 1616*m*, 1590*m*, 1556*s*, 1506*s*, 1461*m*, 1441*s*, 1431*m*, 1416*m*, 1355*w*, 1280*s*, 1258*s*, 1216*m*, 1174*s*, 1159*m*, 1148*s*, 1082*s*, 1071*s*, 1044*s*, 1012*m*, 994*m*, 943*m*, 927*m*, 904*m*, 894*w*, 883*m*, 866*w*, 786*m*, 754*s*, 743*s*, 713*m*, 693*m*, 682*s*, 660*m*; M.p.: 220 °C; HRMS (EI$^+$), [M]$^+$: *m/z* calcd. 365.1086 for $[C_{16}H_{12}O_4S]^+$, found 365.1084; R_f(SiO_2, *i*Hex/EtOAc 7:3) = 0.13.

(Z)-2-(2,6-Dimethoxybenzylidene)benzo[b]thiophen-3(2H)-one 1,1-dioxide (HTI-SO_2 4)

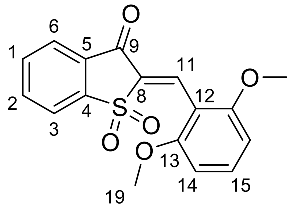

2-(2,6-Dimethoxybenzylidene)benzo[*b*]thiophen-3(2*H*)-one (HTI **4**) (60 mg, 0.20 mmol, 1.0 eq.) was dissolved in acetic acid (2 mL) and H_2O_2 (30%, 0.30 mL, 2.94 mmol, 15.5 eq.) was added. After stirring for 17 h at 23 °C, the reaction was quenched with a saturated aqueous solution of $Na_2S_2O_3$ (10 mL). The aqueous phase was extracted with dichloromethane (3 x 50 mL) and the combined organic phases were dried over Na_2SO_4. After removing the solvent *in vacuo*, the crude product was purified by column chromatography (SiO_2, *i*Hex/EtOAc = 99:1→8:2) and the product HTI-SO_2 **4** was obtained as a yellow solid (22 mg, 0.07 mmol, 35%).

^{1}H NMR (400 MHz, CD_2Cl_2) δ (ppm) = 8.39 (*s*, 1H, H-C(11)), 8.05 (*d*, J = 7.7 Hz, 1H, H-C(6)), 7.96 – 7.92 (*m*, 1H, H-C(3)), 7.89 (*dd*, J = 7.1, 1.2 Hz, 1H, H-C(2)), 7.78 (*ddd*, J = 7.7, 7.1, 1.3 Hz, 1H, H-C(1)), 7.52 (*t*, J = 8.4 Hz, 1H, H-C(15)), 6.64 (*d*, J = 8.4 Hz, 2H, H-C(14)), 3.95 (*s*, 6H, H_3-C(19)); ^{13}C NMR (101 MHz, CD_2Cl_2) δ (ppm) = 181.5 (C(9)), 161.3 (C(13)), 146.9 (C(4)), 136.9 (C(2)), 136.5 (C(8)), 135.7 (C(11)), 133.9 (C(1)), 131.8 (C(5)), 124.8 (C(6)), 121.3 (C(3)), 110.5 (C(12)), 104.0 (C(14), C(15)), 56.4 (C(20)); IR: $\tilde{\nu}$ (cm^{-1}) = 3115*w*, 3025*w*, 2982*w*, 2947*w*, 2842*w*, 1694*m*, 1592*m*, 1563*s*, 1478*s*, 1452*m*, 1431*m*, 1328*w*, 1298*s*, 1284*m*, 1264*s*, 1234*w*, 1202*m*, 1161*w*, 1150*m*, 1121*s*, 1112*s*, 1064*m*, 1045*s*, 1026*m*, 961*w*, 941*s*, 886*w*, 862*w*, 786*s*, 778*m*, 753*s*, 742*s*, 710*w*, 684*s*, 674*m*; M.p.: 181 °C; HRMS (EI$^+$), [M]$^+$: *m/z* calcd. 330.0562 for $[C_{17}H_{14}O_5S]^+$, found 330.0555; R_f (SiO_2, *i*Hex/EtOAc 7:3) = 0.21.

(Z)-2-(2,4,6-Trimethylbenzylidene)benzo[b]thiophen-3(2H)-one 1,1-dioxide (HTI-SO_2 5)

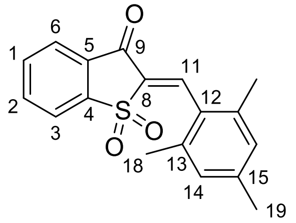

2-(2,4,6-Trimethylbenzylidene)benzo[*b*]thiophen-3(2*H*)-one (HTI **5**) (30 mg, 0.11 mmol, 1.0 eq.) was dissolved in conc. acetic acid (1 mL) and hydrogen peroxide (30%, 0.15 mL, 1.47 mmol, 13.3 eq.) was added. After stirring for 23 h at 23 °C, the reaction was quenched with a saturated aqueous solution of

$Na_2S_2O_3$ (10 mL). The aqueous phase was extracted with dichloromethane (3 x 20 mL) and the combined organic phases were dried over Na_2SO_4. After removing the solvent *in vacuo*, the crude product was purified by column chromatography (SiO_2, *i*Hex/EtOAc = 99:1→7:3) and the product HTI-SO_2 **5** was obtained as a yellow solid (4 mg, 0.01 mmol, 5%).

^{1}H NMR (400 MHz, CD_2Cl_2) δ (ppm) = 8.32 (*s*, 1H, H-C(11)), 8.13 (*dt*, *J* = 7.7, 1.0 Hz, 1H, H-C(6)), 7.95 (*dt*, *J* = 4.2, 0.8 Hz, 2H, H-C(2), H-C(3)), 7.86 (*dt*, *J* = 7.8, 4.2 Hz, 1H, H-C(1)), 6.96 (*s*, 2H, H_2-C(14)), 2.33 (*s*, 3H, H_3-C(19)), 2.25 – 2.22 (*m*, 6H, H_3-C(18)); ^{13}C NMR (101 MHz, CD_2Cl_2) δ (ppm) = 178.7 (C(9)), 147.2 (C(11)), 145.7 (C(4)), 139.7 (C(15)), 137.7 (C(2)), 135.9 (C(13)), 134.8 (C(1)), 132.6 (C(5)), 128.9 (C(8/12)), 128.6 (C(14)), 128.0 (C(8/12)), 125.5 (C(6)), 121.9 (C(3)), 21.43 (C(19)), 20.40 (C(18)); IR: $\tilde{\nu}$ (cm^{-1}) = 2978*w*, 2922*w*, 2856*w*, 1710*m*, 1625*m*, 1585*w*, 1450*w*, 1380*w*, 1326*w*, 1296*s*, 1274*m*, 1214*w*, 1203*m*, 1149*s*, 1123*w*, 1066*w*, 1050*w*, 918*w*, 844*w*, 794*w*, 754*s*, 746*s*, 711*w*, 988*s*, 666*w*; M.p.: 251 °C; HRMS (EI^+), $[M]^+$: *m/z* calcd. 312.0820 for $[C_{18}H_{16}O_3S]^+$, found 312.0812; R_f(SiO_2, *i*Hex/EtOAc 7:3) = 0.46.

Thermal Isomerization

The *Z* isomers are the thermodynamically most stable forms of all switches. At elevated temperatures the *E* isomers are interconverting into the *Z* isomers. The first-order rate constants for the thermal *E* to *Z* isomerization were determined from the decay kinetics of the *Z* isomers either at 23 °C or during heating. In case the thermal isomerizations do not proceed to 100% in one direction but are instead dynamic equilibria the observed decays are composites of both isomerization processes (*E* to *Z* and vice versa) and have to be analyzed as shown in the literature.[5]

Table S1 Quantitative Comparison of free activation enthalpies $\Delta G^{‡}$. Thermal *E* to *Z* decays were measured in toluene-d_8. * Value represents the average of two independent measurements.

HTI	$\Delta G^{‡}$ (therm. *E*/*Z*) /kcal mol^{-1}
**1**	30.9
SO **1**	29.7
SO_2 **1**	28.2
**2**	26.4
SO **2**	30.9
SO_2 **2**	29.4
**3**	21.4
SO **3**	22.8
SO_2 **3**	-
**4**	31.7*
SO **4**	23.3
SO_2 **4**	24.7
**5**	33.0
SO **5**	>33.0
SO_2 **5**	>33.0

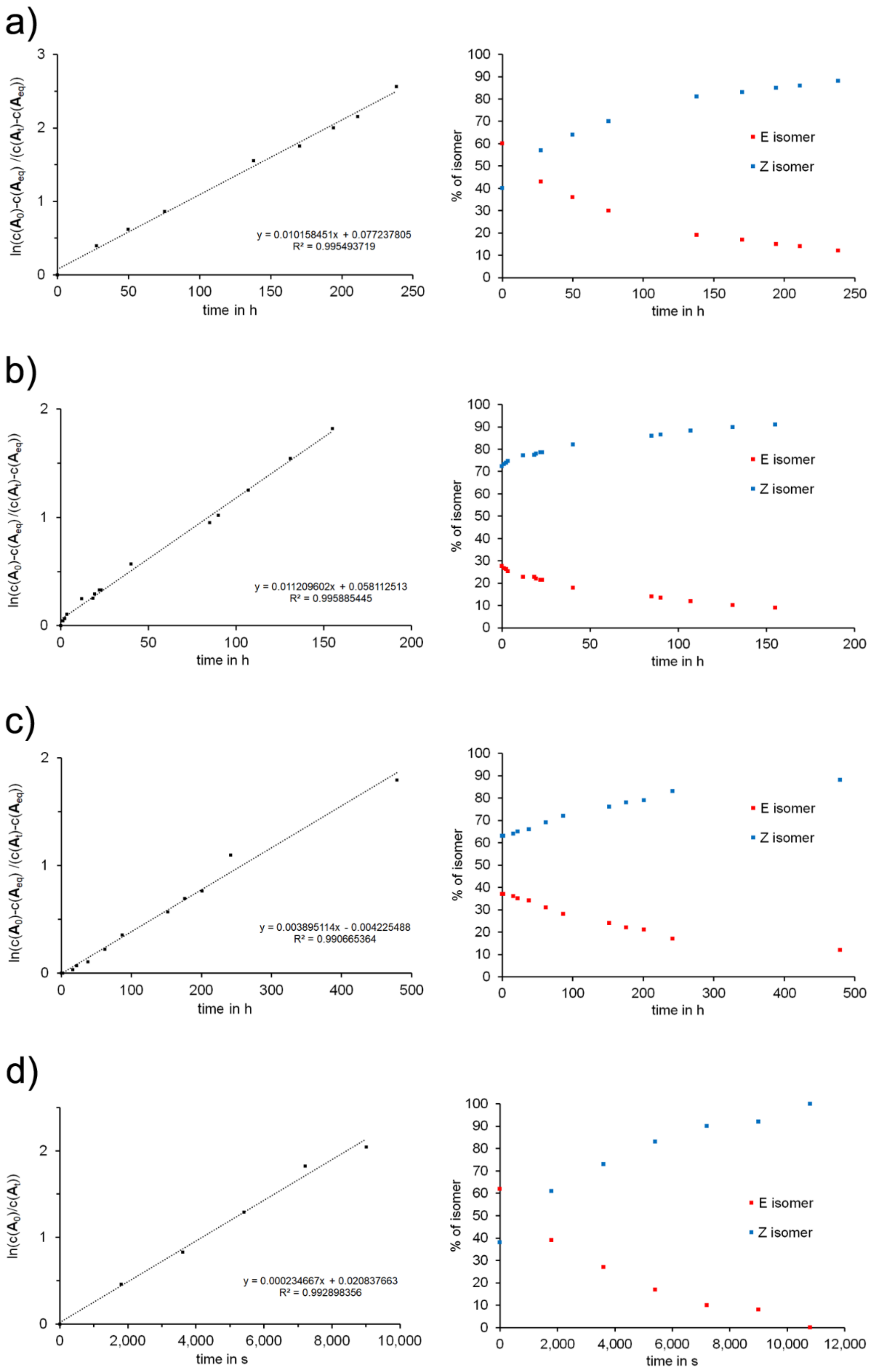

Figure S1 First order kinetic analysis of thermal *E* to *Z* isomerization (left) and decrease of the *E* isomer and increase of the *Z* isomer with time (right) in toluene-d_8. a) HTI **4** at 100 °C. b) HTI-SO **1** at 80 °C. c) HTI-SO **2** at 85 °C. d) HTI-SO **3** at 25 °C.

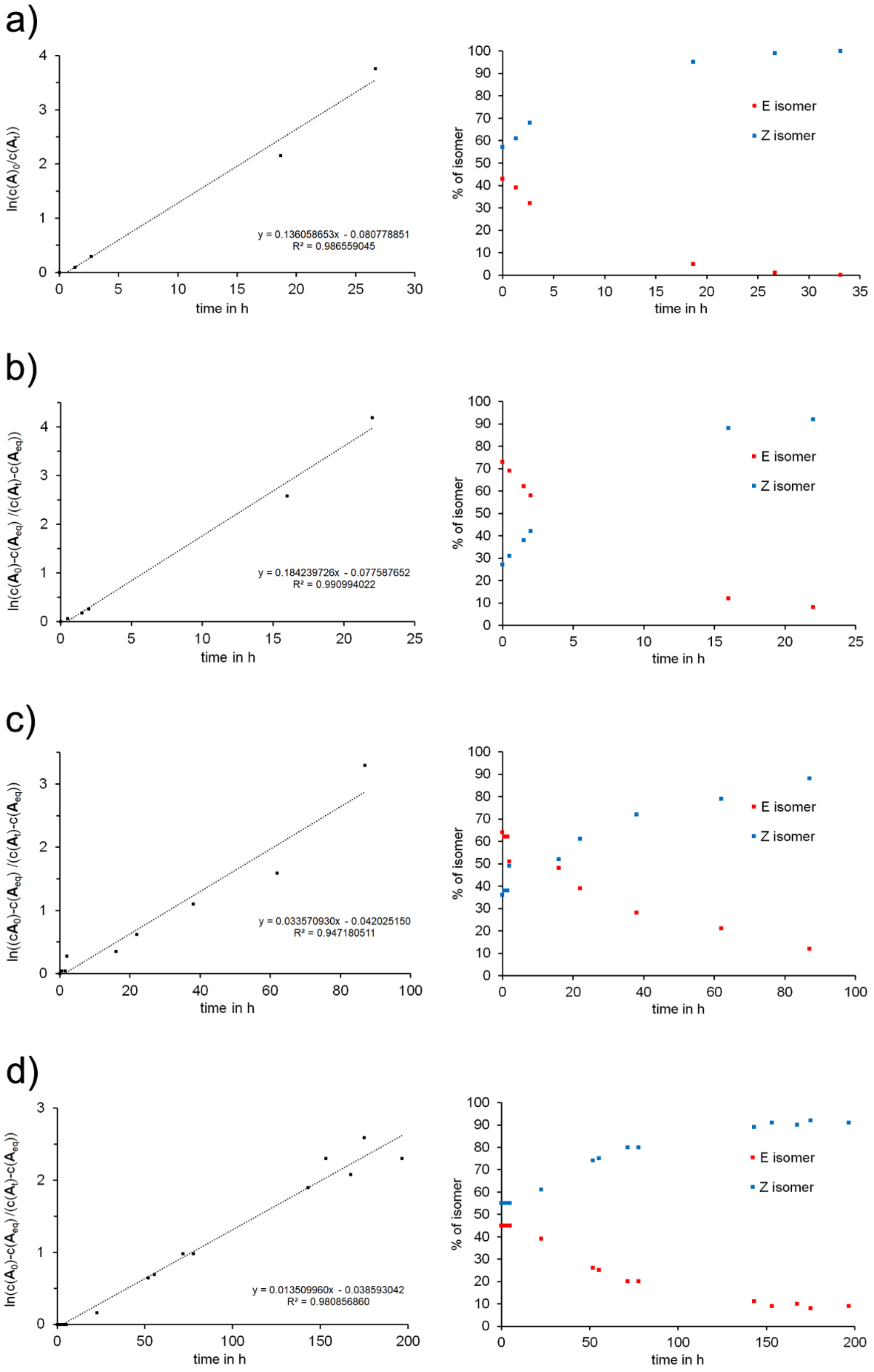

Figure S2 First order kinetic analysis of thermal *E* to *Z* isomerization (left) and decrease of the *E* isomer and increase of the *Z* isomer with time (right) in toluene-d_8. a) HTI-SO **4** at 85 °C. b) HTI-SO_2 **1** at 85 °C. c) HTI-SO_2 **2** at 85 °C. d) HTI-SO_2 **4** at 23 °C.

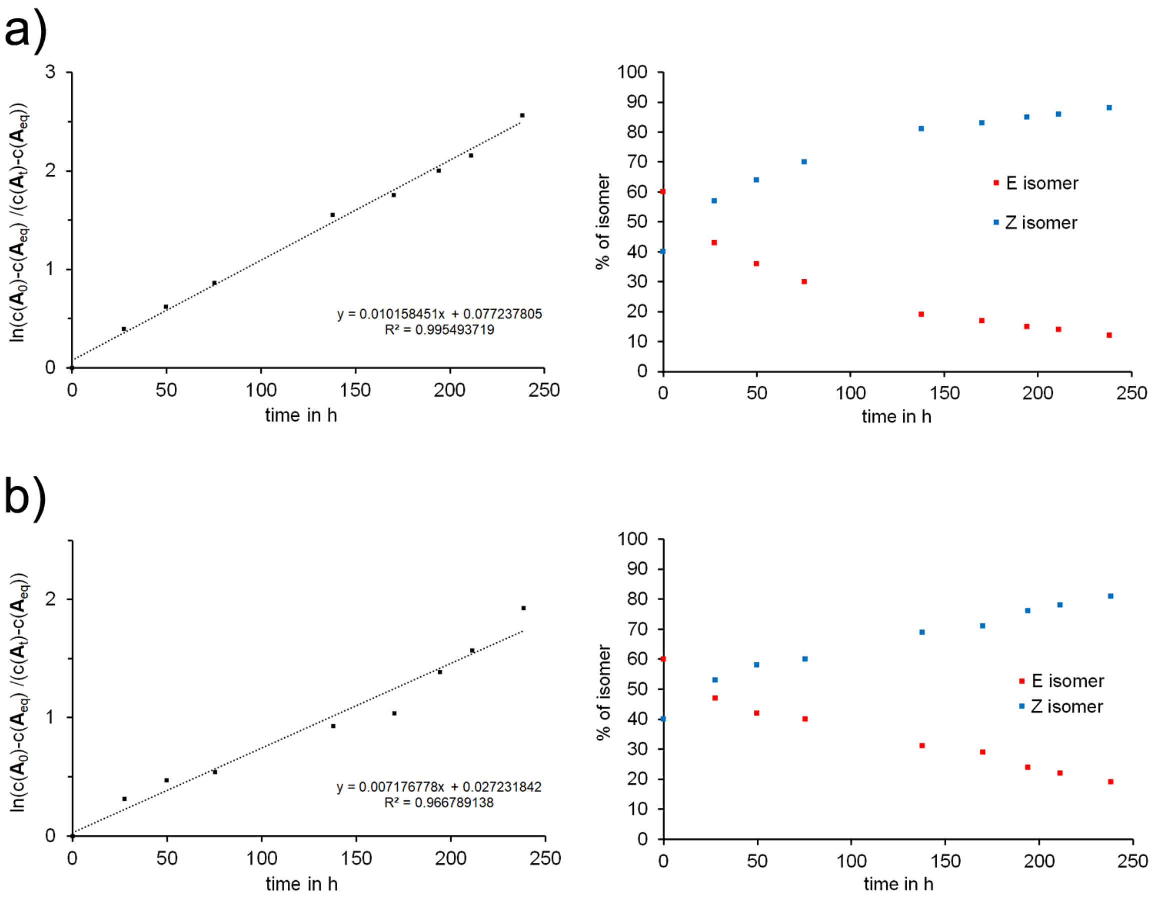

Figure S3 Comparison of two independent measurements of the first order kinetics associated with thermal *E* to *Z* isomerization (left) and decrease of the *E* isomer and increase of the *Z* isomer with time (right) in toluene-d_8 at 100 °C. a) First experiment. b) Second experiment.

Table S2 Comparison of free activation enthalpies $\Delta G^{\ddagger}$ for thermal *E* to *Z* isomerization of parent HTI **4** measured in two independent experiments at 100 °C in toluene-d_8. Experiment a) and b) refer to the measured data represented in Figure 3a and 3b, respectively. Only small deviations in the range of 0.3 kcal/mol are observed.

experiment	$\Delta G^{\ddagger}$ (therm. *E*/*Z*) /kcal mol^{-1}
a)	31.6
b)	31.9

Photophysical Properties

a)

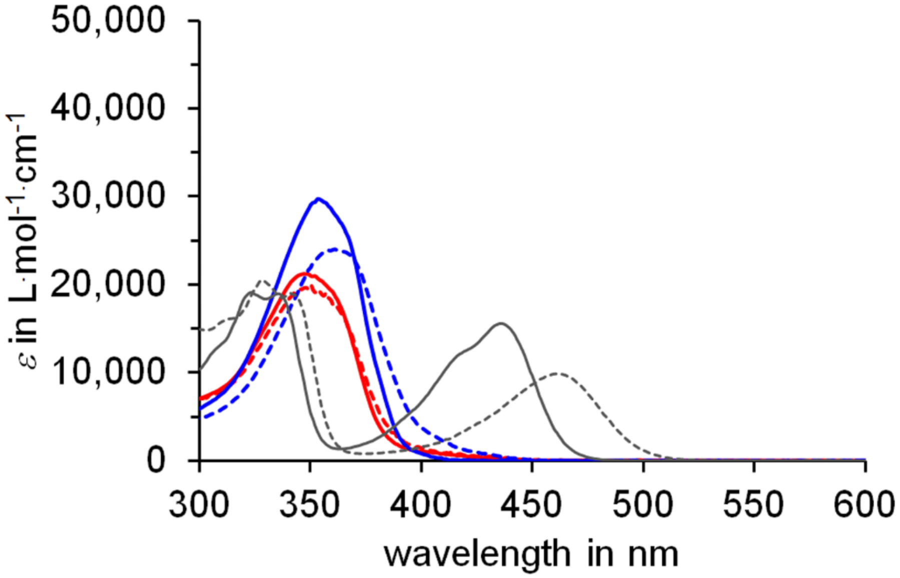

b)

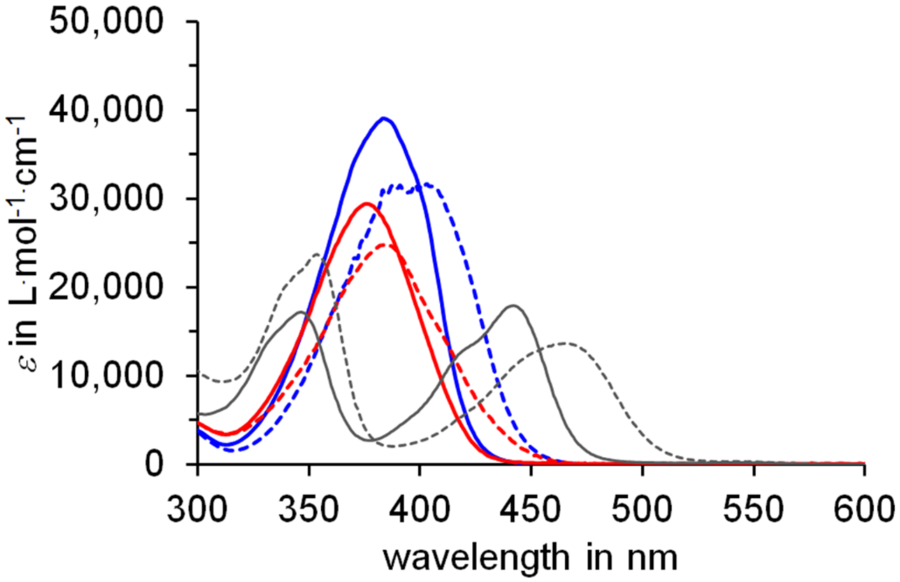

Figure S4 Molar absorption coefficient *ε* at 23 °C of a) parent HTI **1** (grey, *Z* isomer solid, *E* isomer dashed), HTI-SO **1** (red, *Z* isomer solid, *E* isomer dashed) and HTI-SO_2 **1** (blue, *Z* isomer solid, *E* isomer dashed) in toluene. b) parent HTI **2** (grey, *Z* isomer solid, *E* isomer dashed), HTI-SO **2** (red, *Z* isomer solid, *E* isomer dashed) and HTI-SO_2 **2** (blue, *Z* isomer solid, *E* isomer dashed) in CH_2Cl_2.

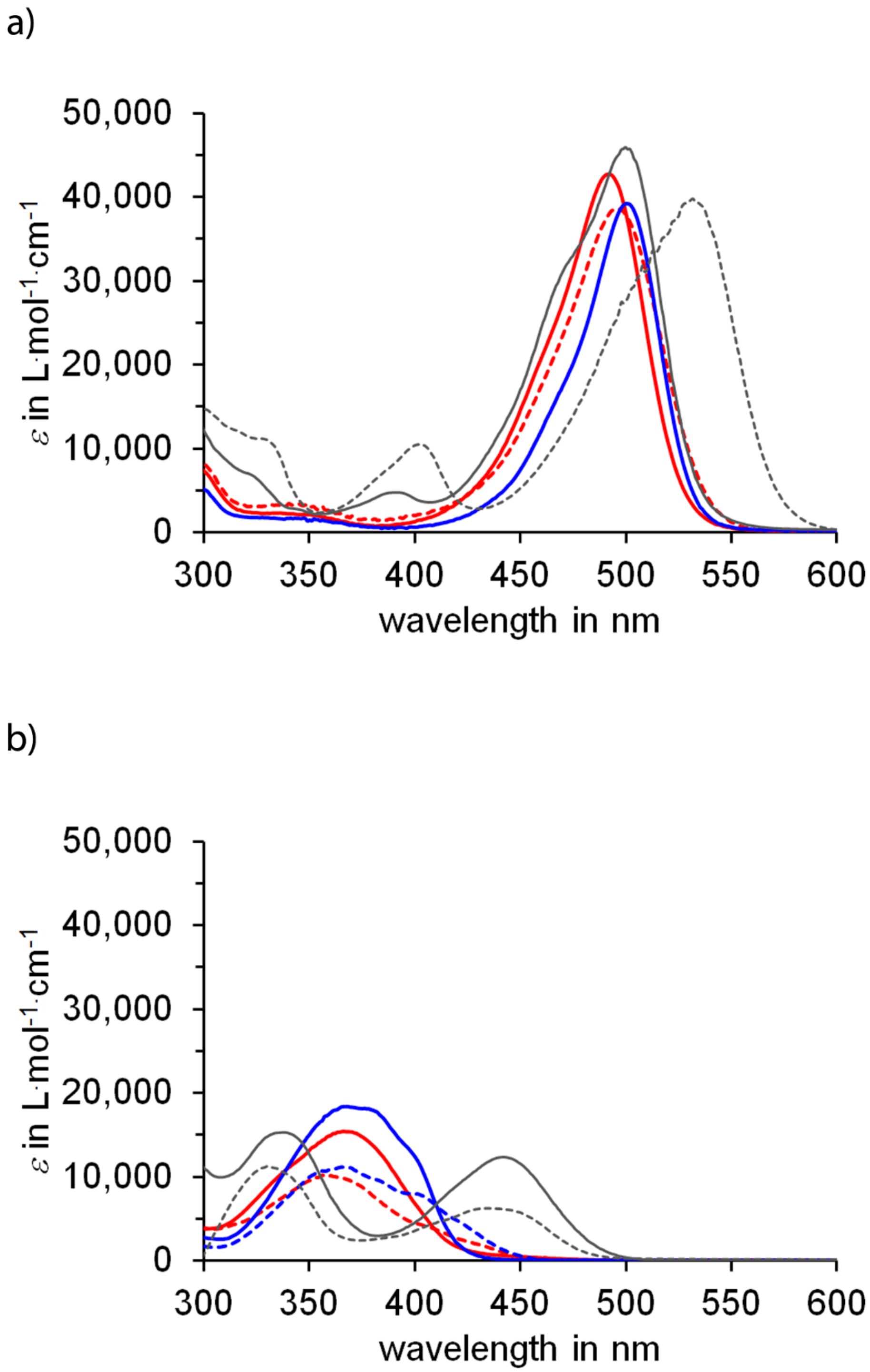

Figure S5 Molar absorption coefficient ε at 23 °C of a) parent HTI **3** (grey, *Z* isomer solid, *E* isomer dashed), HTI-SO **3** (red, *Z* isomer solid, *E* isomer dashed) and HTI-SO_2 **3** (blue, *Z* isomer solid, *E* isomer dashed) in toluene. b) parent HTI **4** (grey, *Z* isomer solid, *E* isomer dashed), HTI-SO **4** (red, *Z* isomer solid, *E* isomer dashed) and HTI-SO_2 **4** (blue, *Z* isomer solid, *E* isomer dashed) in CD_2Cl_2.

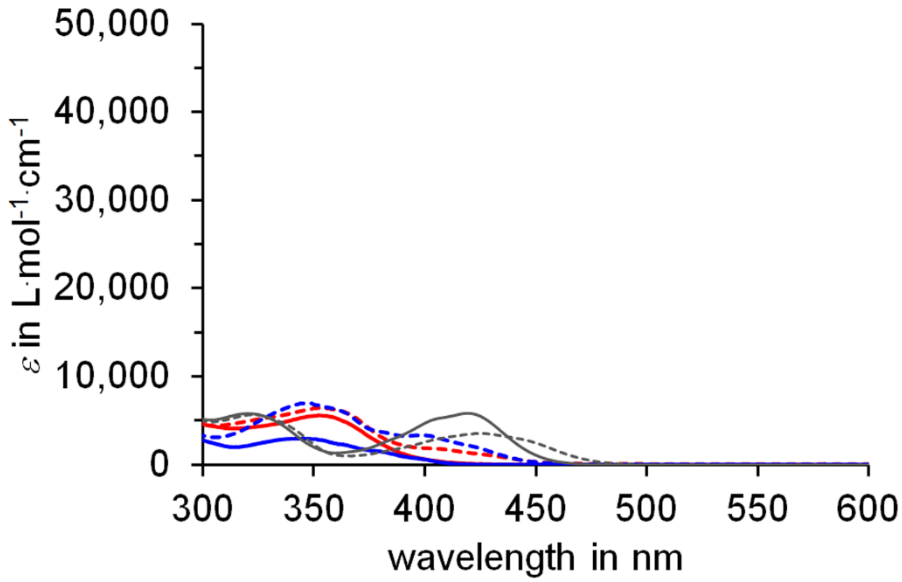

Figure S6 Molar absorption coefficient ε of parent HTI **5** (grey, *Z* isomer solid, *E* isomer dashed), HTI-SO **5** (red, *Z* isomer solid, *E* isomer dashed) and HTI-SO_2 **5** (black, *Z* isomer solid, *E* isomer dashed) in CH_2Cl_2 at 23 °C.

Solvatochromic effects

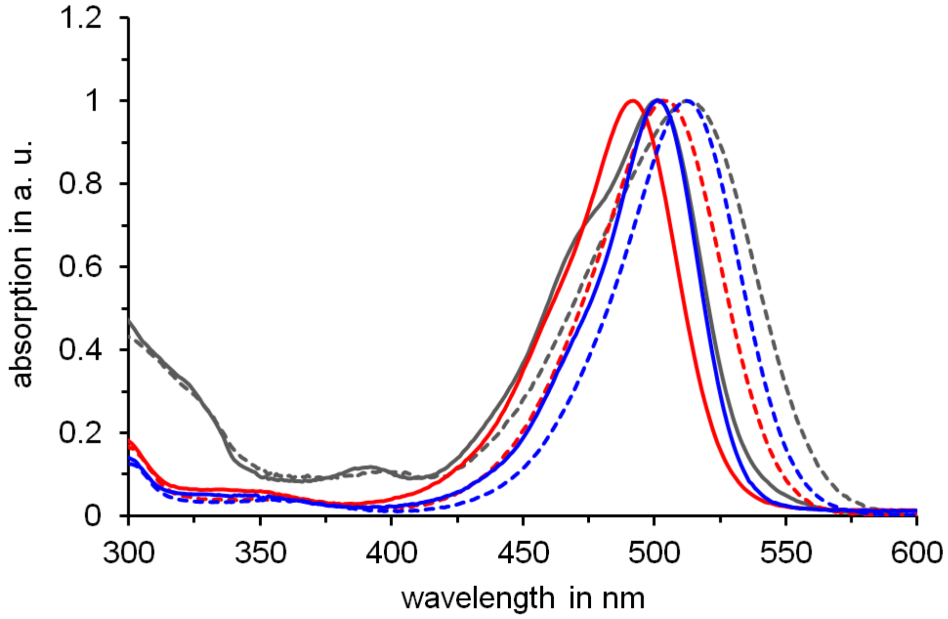

Figure S7 Solvatochromic effects (normalized) of parent HTI **3** (grey), HTI-SO **3** (red) and HTI-SO_2 **3** (blue) in CH_2Cl_2 (dashed) and toluene (solid) at 23 °C.

Table S3 Quantitative comparison of the solvatochromic effects of *Z* isomers of HTI series **3** in CH_2Cl_2 and toluene.

HTI	λ_{max} in CH_2Cl_2 of most redshifted abs. /nm	λ_{max} in toluene of most redshifted abs. /nm
**3**	500	512
SO **3**	492	504
SO_2 **3**	501	512

Photoswitching

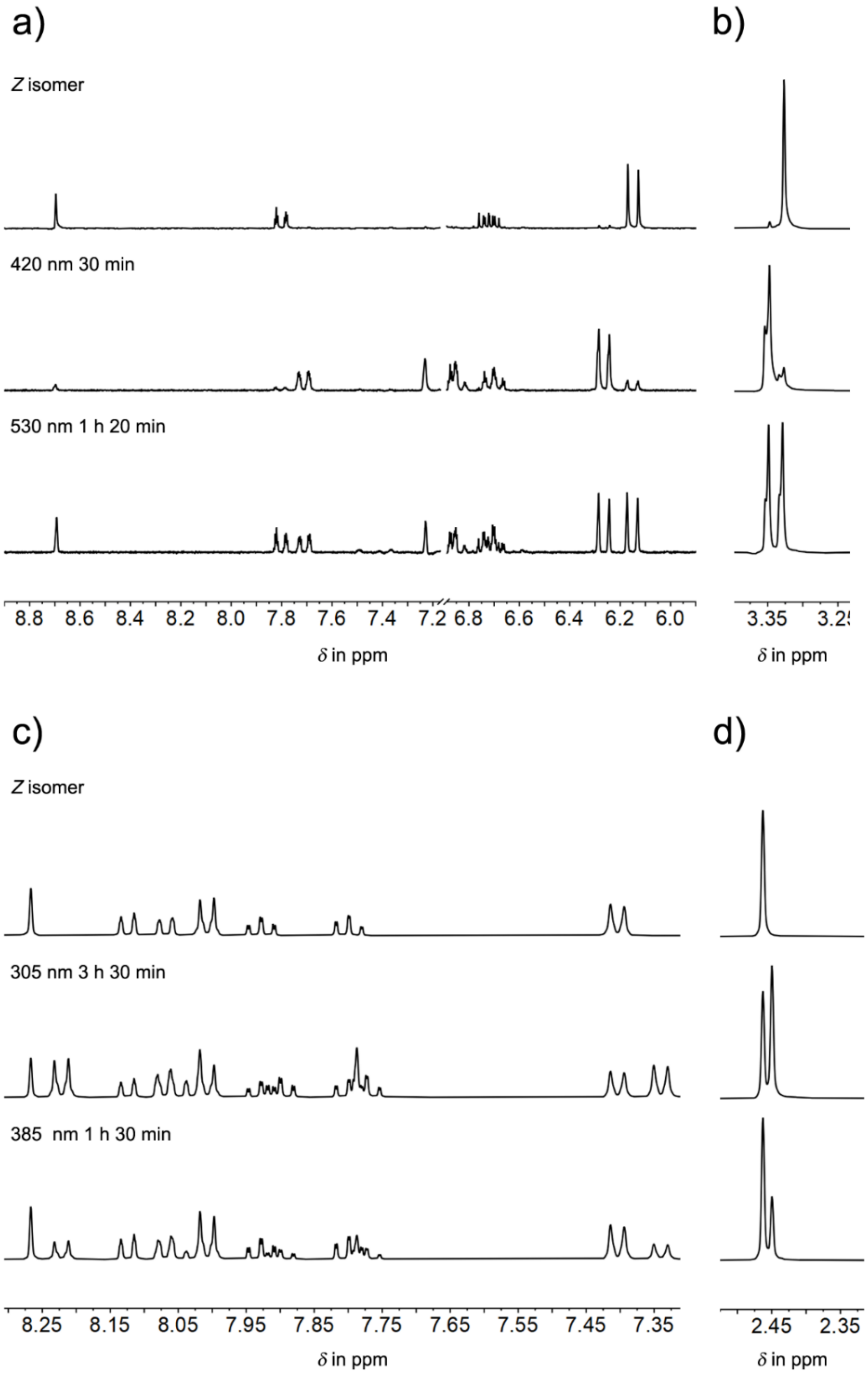

Figure S8 ^{1}H NMR spectra of HTI **4** and HTI-SO **1** before and after irradiation. a) Aromatic region of the spectra of HTI **4** (400 MHz, toluene-*d*$_8$, 25 °C). b) Aliphatic region of the spectra (400 MHz, toluene-*d*$_8$, 25 °C). c) Aromatic region of the spectra of HTI-SO **1** (400 MHz, CD_2Cl_2, 25 °C). d) Aliphatic region of the spectra of HTI-SO **1** (400 MHz, CD_2Cl_2, 25 °C). Signal magnification varies between aromatic and aliphatic regions.

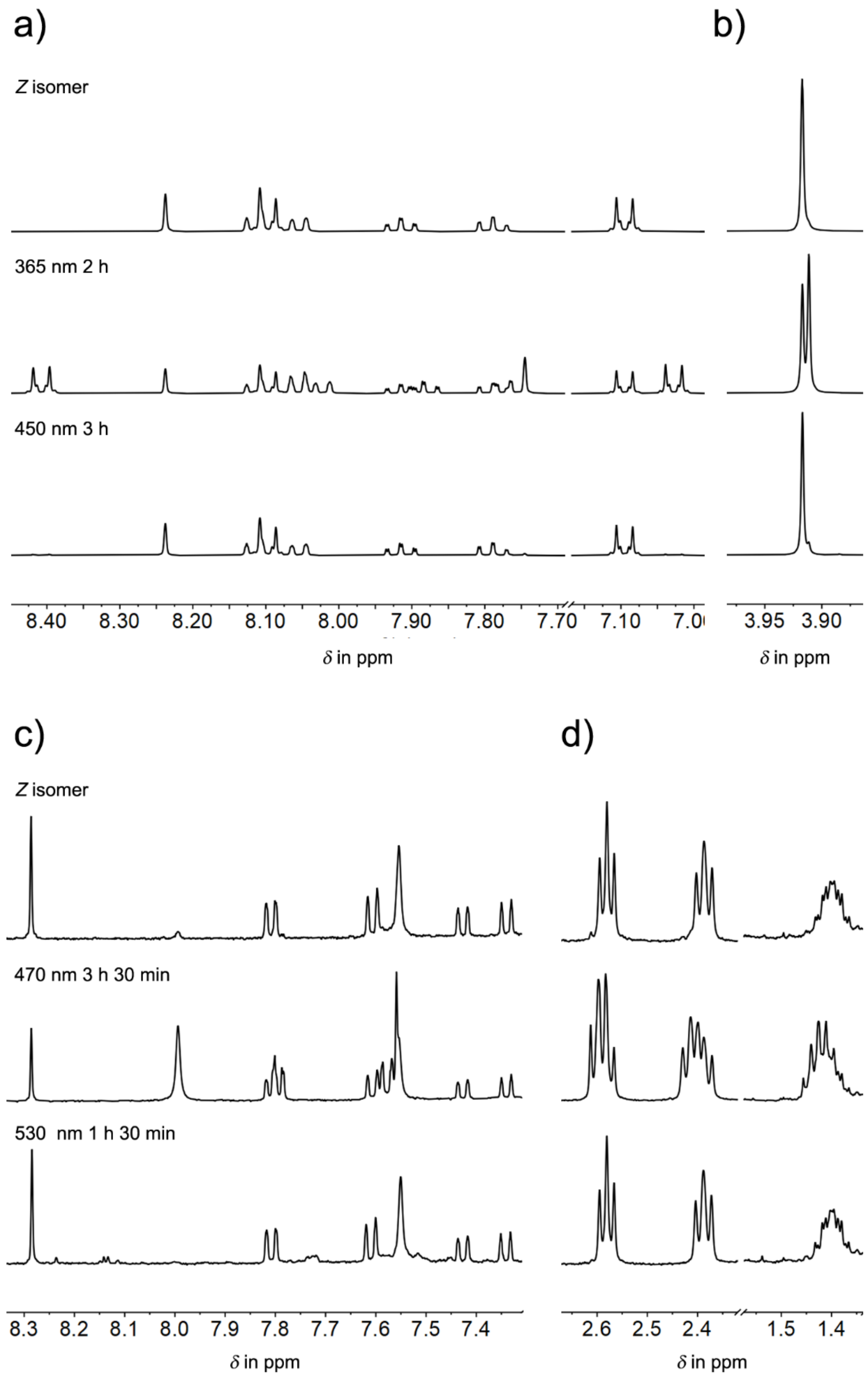

Figure S9 ^{1}H NMR spectra of HTI-SO **2** and HTI-SO **3** before and after irradiation. a) Aromatic region of the spectra of HTI-SO **2** (400 MHz, CD_2Cl_2, 25 °C). b) Aliphatic region of the spectra of HTI-SO **2** (400 MHz, CD_2Cl_2, 25 °C). c) Aromatic region of the spectra of HTI-SO **3** (400 MHz, toluene-d_8, 25 °C). d) Aliphatic region of the spectra of HTI-SO **3** (400 MHz, toluene-d_8, 25 °C). Signal magnification varies between aromatic and aliphatic regions.

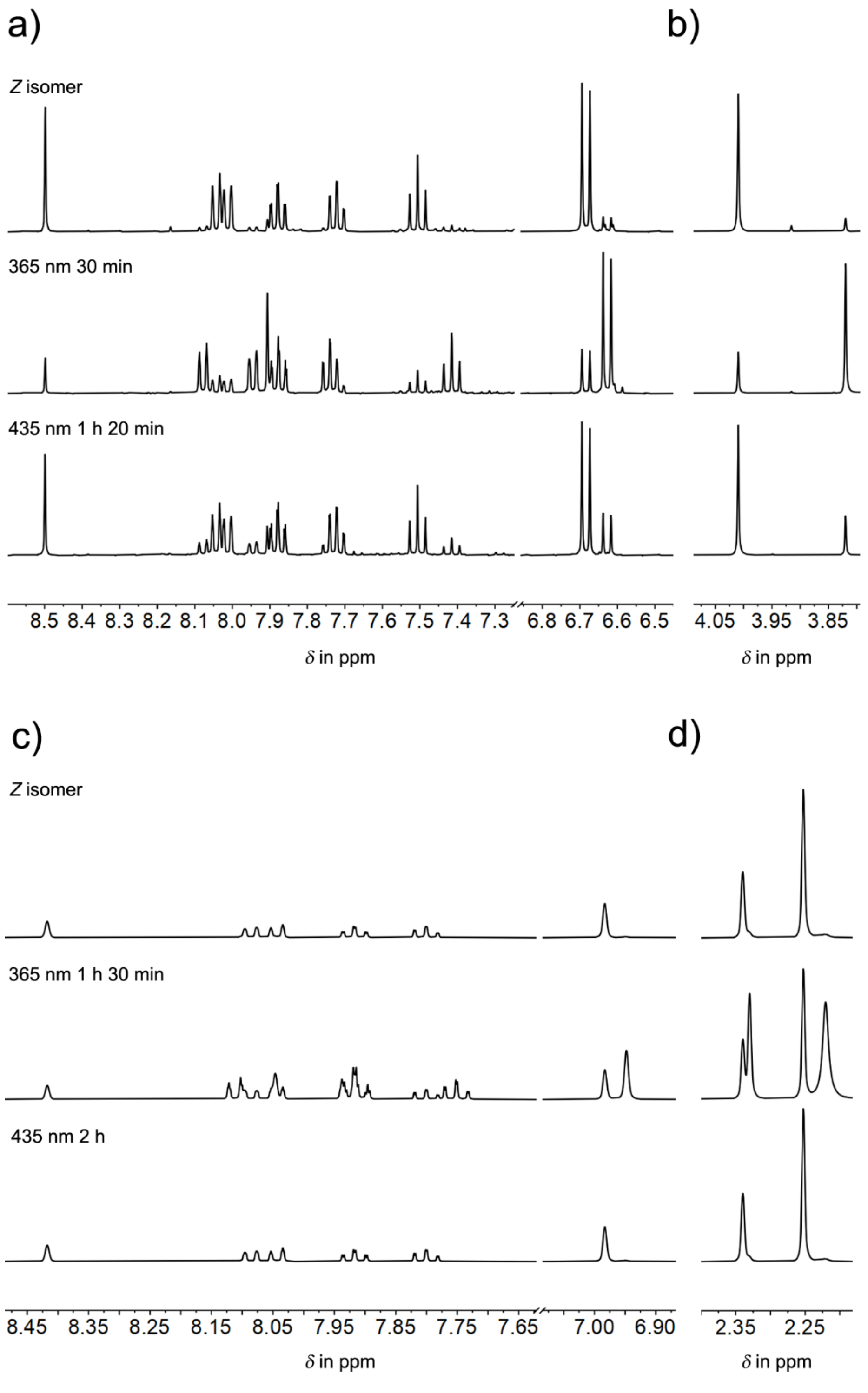

Figure S10 ^{1}H NMR spectra of HTI-SO **4** and HTI-SO **5** before and after irradiation. a) Aromatic region of the spectra of HTI-SO **4** (400 MHz, CD_2Cl_2, 25 °C). b) Aliphatic region of the spectra of HTI-SO **4** (400 MHz, CD_2Cl_2, 25 °C). c) Aromatic region of the spectra of HTI-SO **5** (400 MHz, CD_2Cl_2, 25 °C). d) Aliphatic region of the spectra of HTI-SO **5** (400 MHz, CD_2Cl_2, 25 °C). Signal magnification varies between aromatic and aliphatic regions.

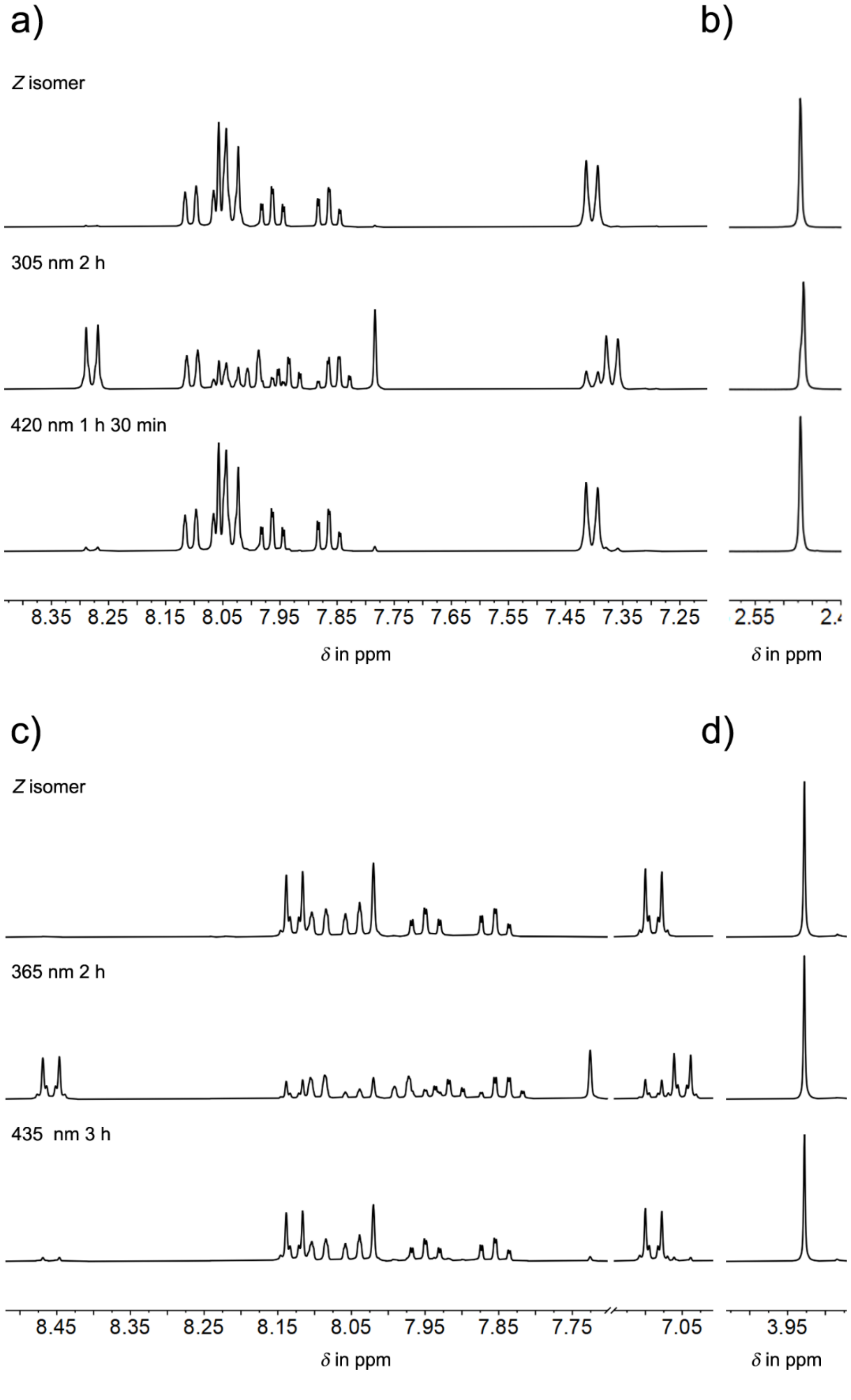

Figure S11 ^{1}H NMR spectra of HTI-SO_2 **1** and HTI-SO_2 **2** before and after irradiation. a) Aromatic region of the spectra of HTI-SO_2 **1** (400 MHz, CD_2Cl_2, 25 °C). b) Aliphatic region of the spectra of HTI-SO_2 **1** (400 MHz, CD_2Cl_2, 25 °C). c) Aromatic region of the spectra of HTI-SO_2 **2** (400 MHz, CD_2Cl_2, 25 °C). d) Aliphatic region of the spectra of HTI-SO_2 **2** (400 MHz, CD_2Cl_2, 25 °C). Signal magnification varies between aromatic and aliphatic regions.

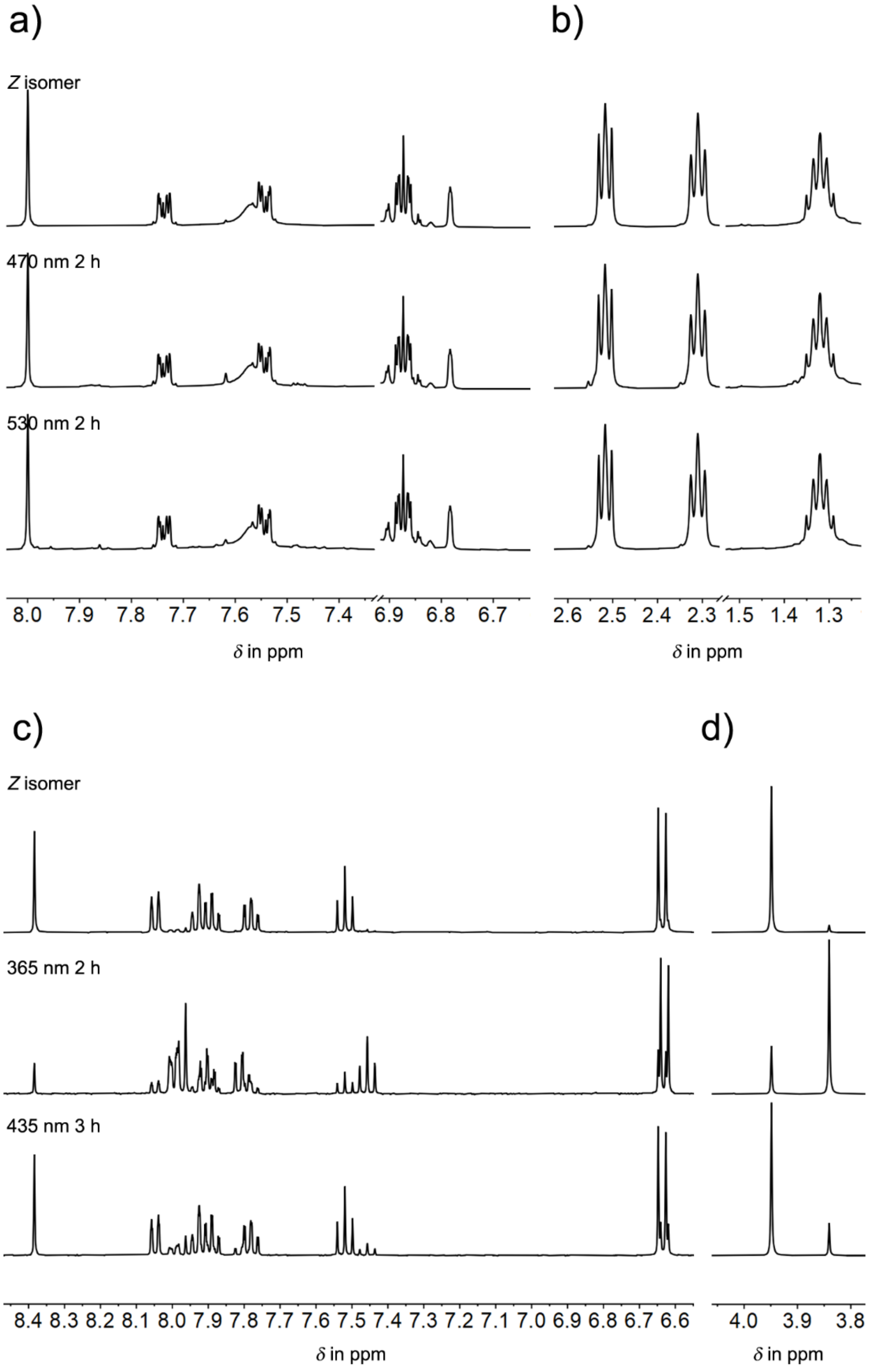

Figure S12 ^{1}H NMR spectra of HTI-SO_2 **3** and HTI-SO_2 **4** before and after irradiation. a) Aromatic region of the spectra of HTI-SO_2 **3** (400 MHz, toluene-d_8, 25 °C). b) Aliphatic region of the spectra of HTI-SO_2 **3** (400 MHz, CD_2Cl_2, 25 °C). c) Aromatic region of the spectra of HTI-SO_2 **4** (400 MHz, CD_2Cl_2, 25 °C). d) Aliphatic region of the spectra of HTI-SO_2 **4** (400 MHz, CD_2Cl_2, 25 °C). Signal magnification varies between aromatic and aliphatic regions.

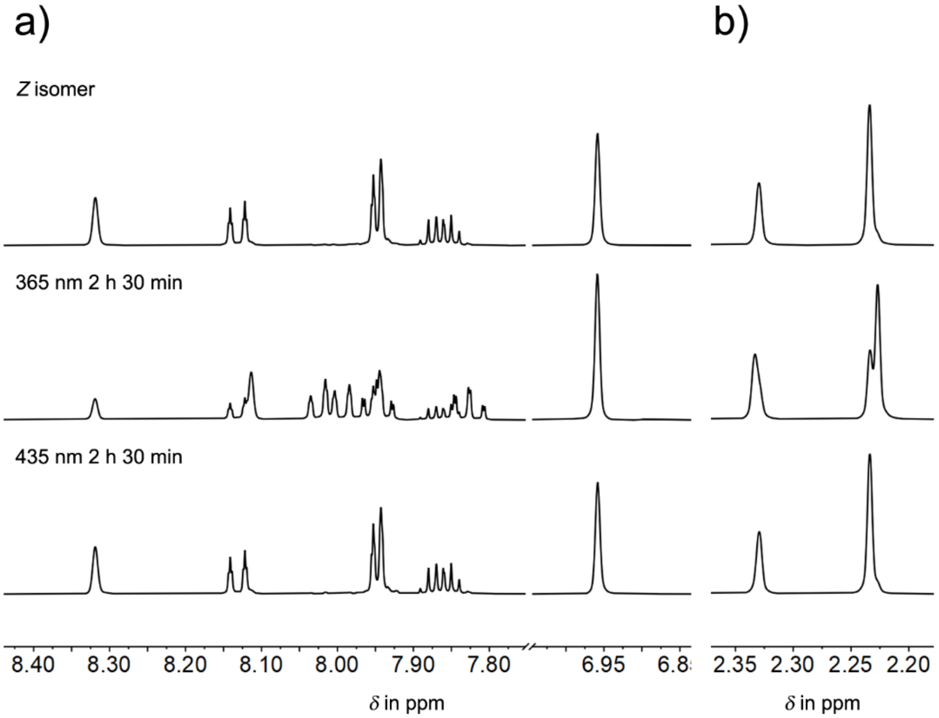

Figure S13 ^1H NMR spectra of HTI-SO_2 **5** before and after irradiation. a) Aromatic region of the spectra of HTI-SO_2 **5** (400 MHz, CD_2Cl_2, 25 °C). b) Aliphatic region of the spectra of HTI-SO_2 **5** (400 MHz, CD_2Cl_2, 25 °C). Signal magnification varies between aromatic and aliphatic regions.

NMR Spectra

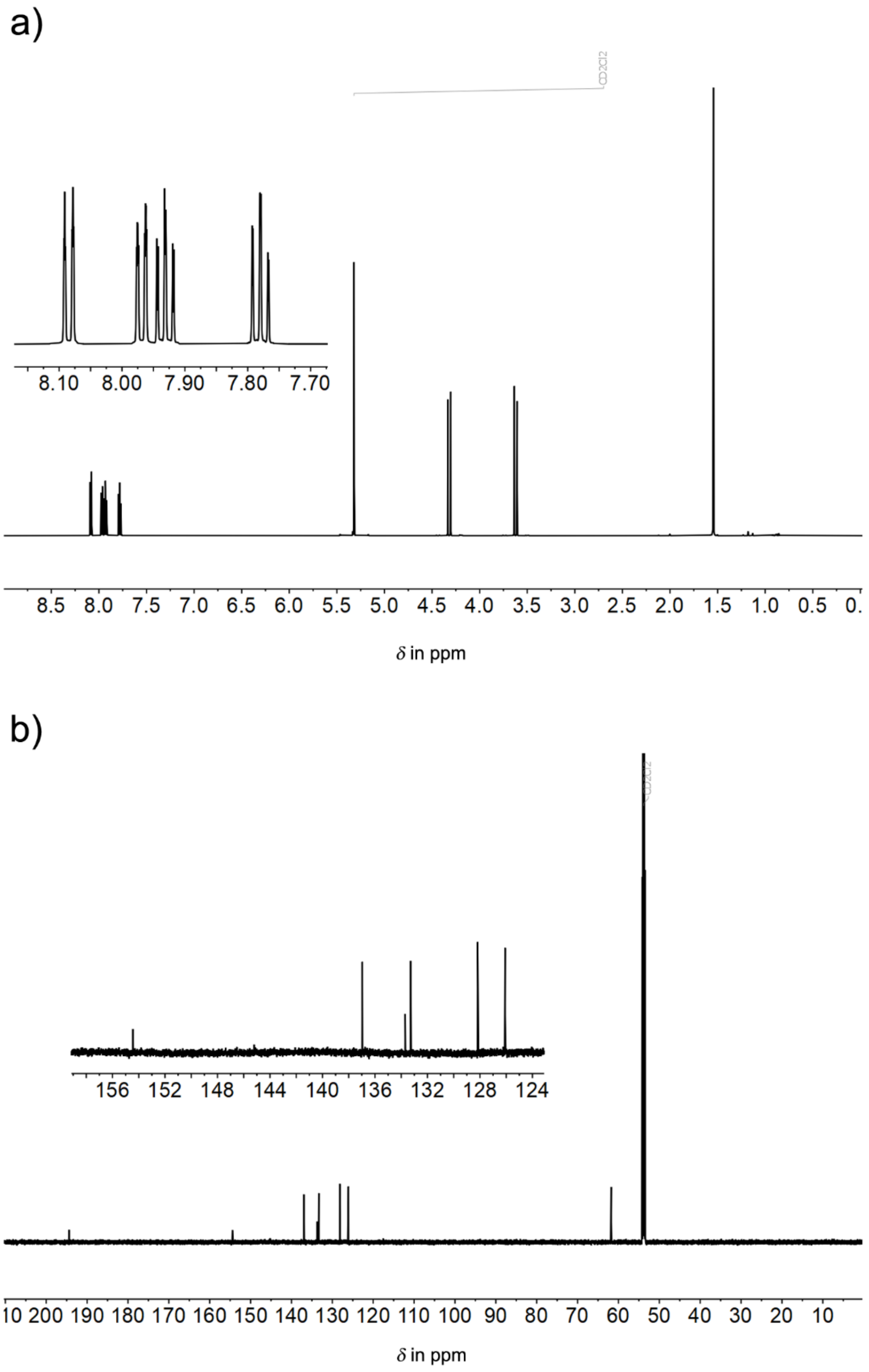

Figure S14 NMR spectra of benzo[*b*]thiophen-3(2*H*)-one 1-oxide (**6**) in CD_2Cl_2 at 23 °C. a) ^{1}H NMR spectrum (400 MHz). b) ^{13}C NMR spectrum (101 MHz).

a)

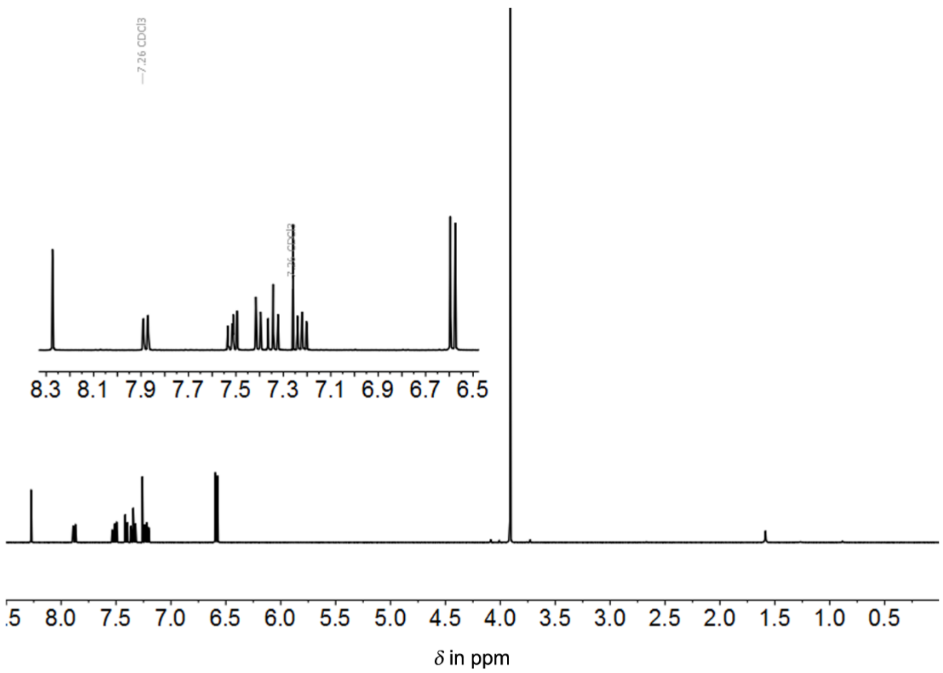

b)

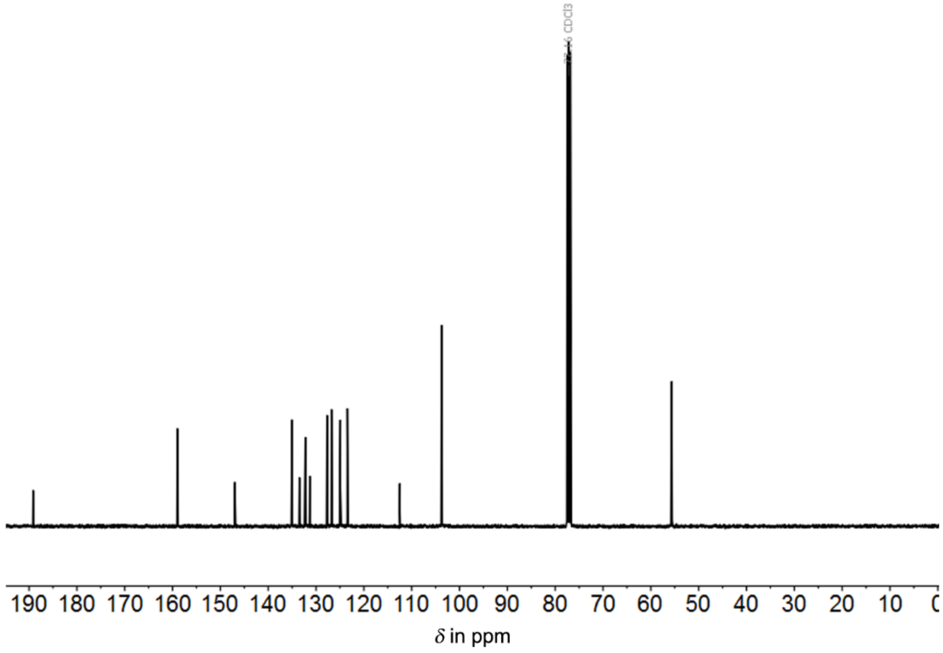

Figure S15 NMR spectra of parent HTI **4** in $CDCl_3$ at 23 °C. a) ^{1}H NMR spectrum (400 MHz). b) ^{13}C NMR spectrum (101 MHz).

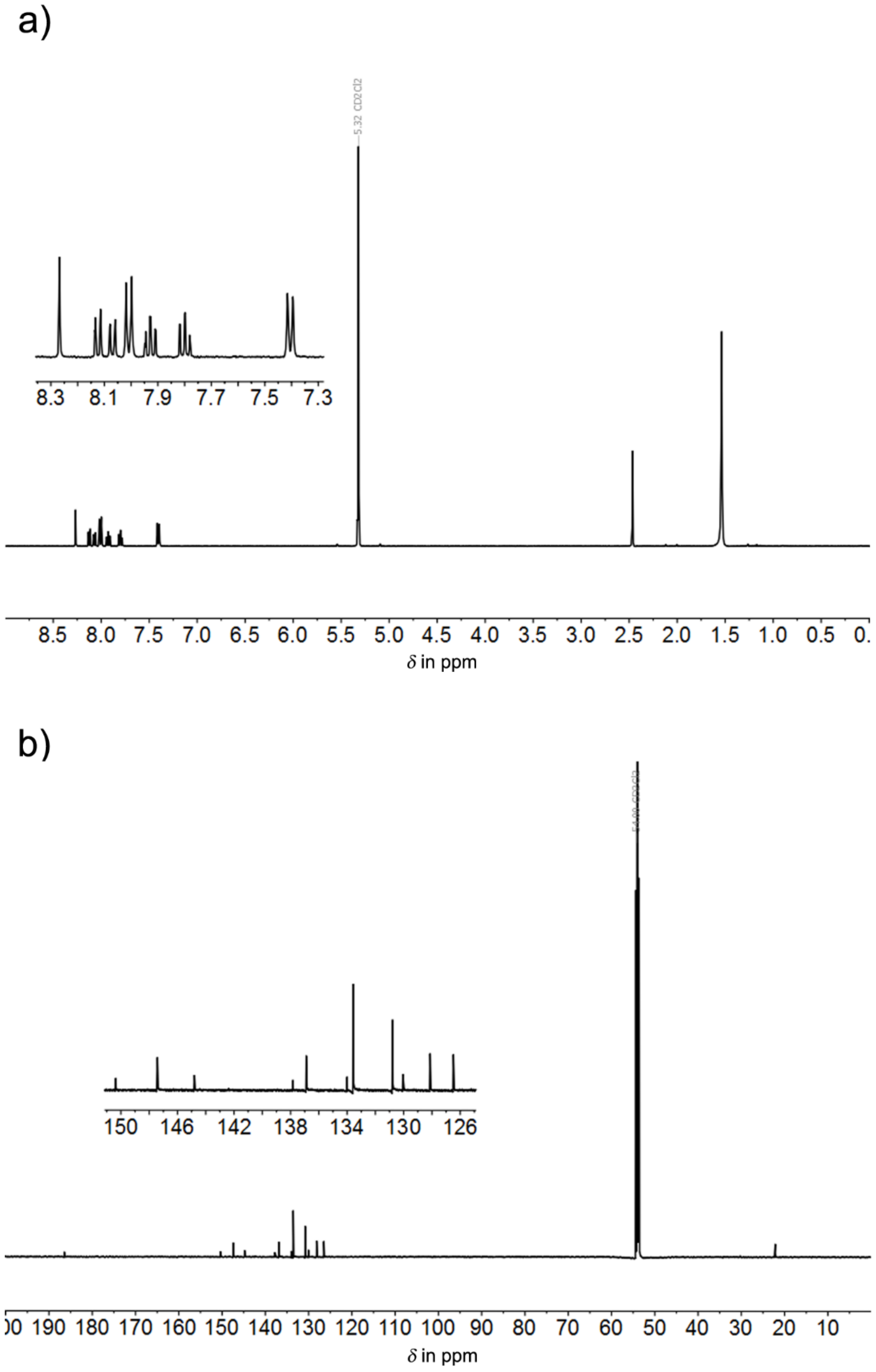

Figure S16 NMR spectra of HTI-SO **1** in CD_2Cl_2 at 23 °C. a) ^{1}H NMR spectrum (600 MHz). b) ^{13}C NMR spectrum (150 MHz).

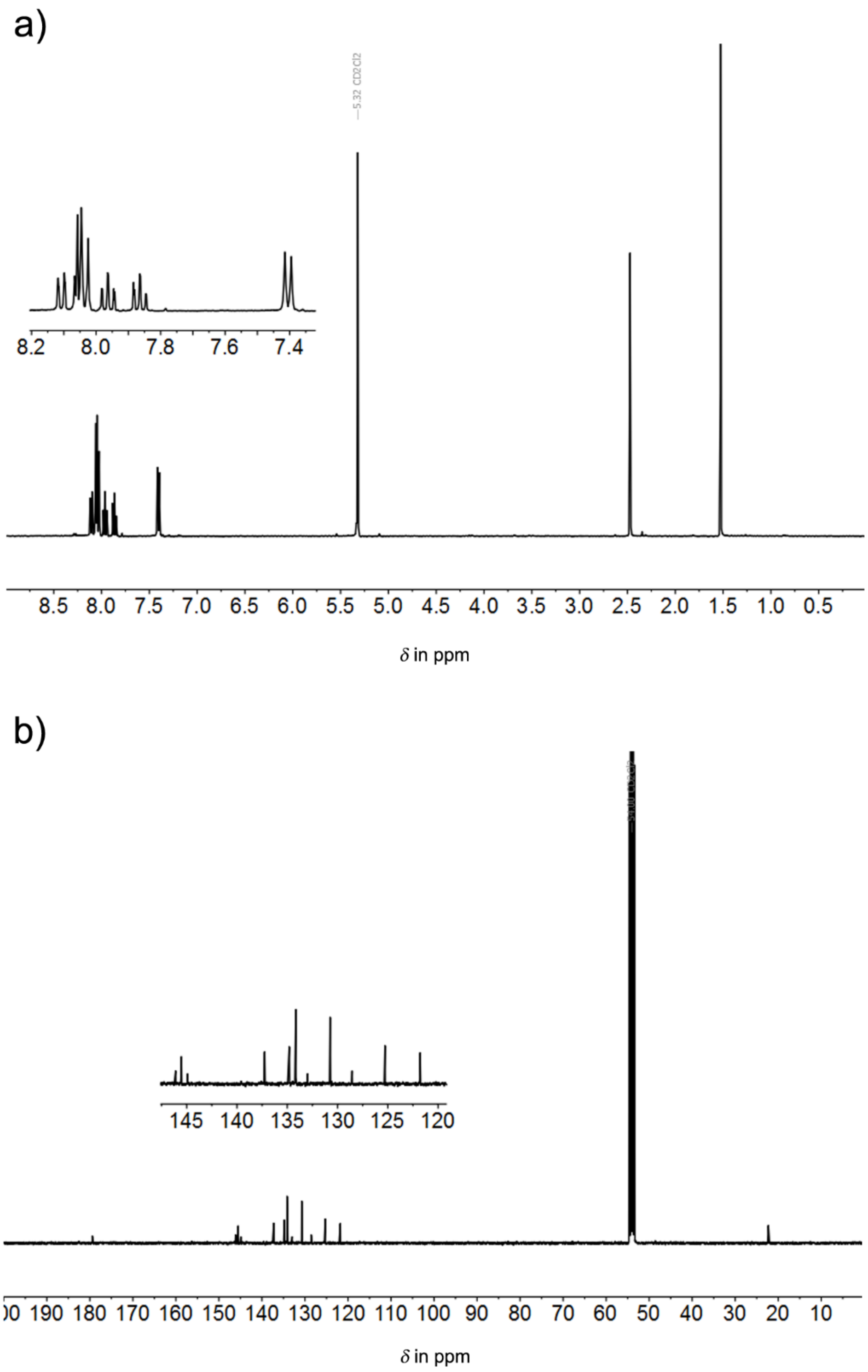

Figure S17 NMR spectra of HTI-SO_2 **1** in CD_2Cl_2 at 23 °C. a) ^{1}H NMR spectrum (600 MHz). b) ^{13}C NMR spectrum (101 MHz).

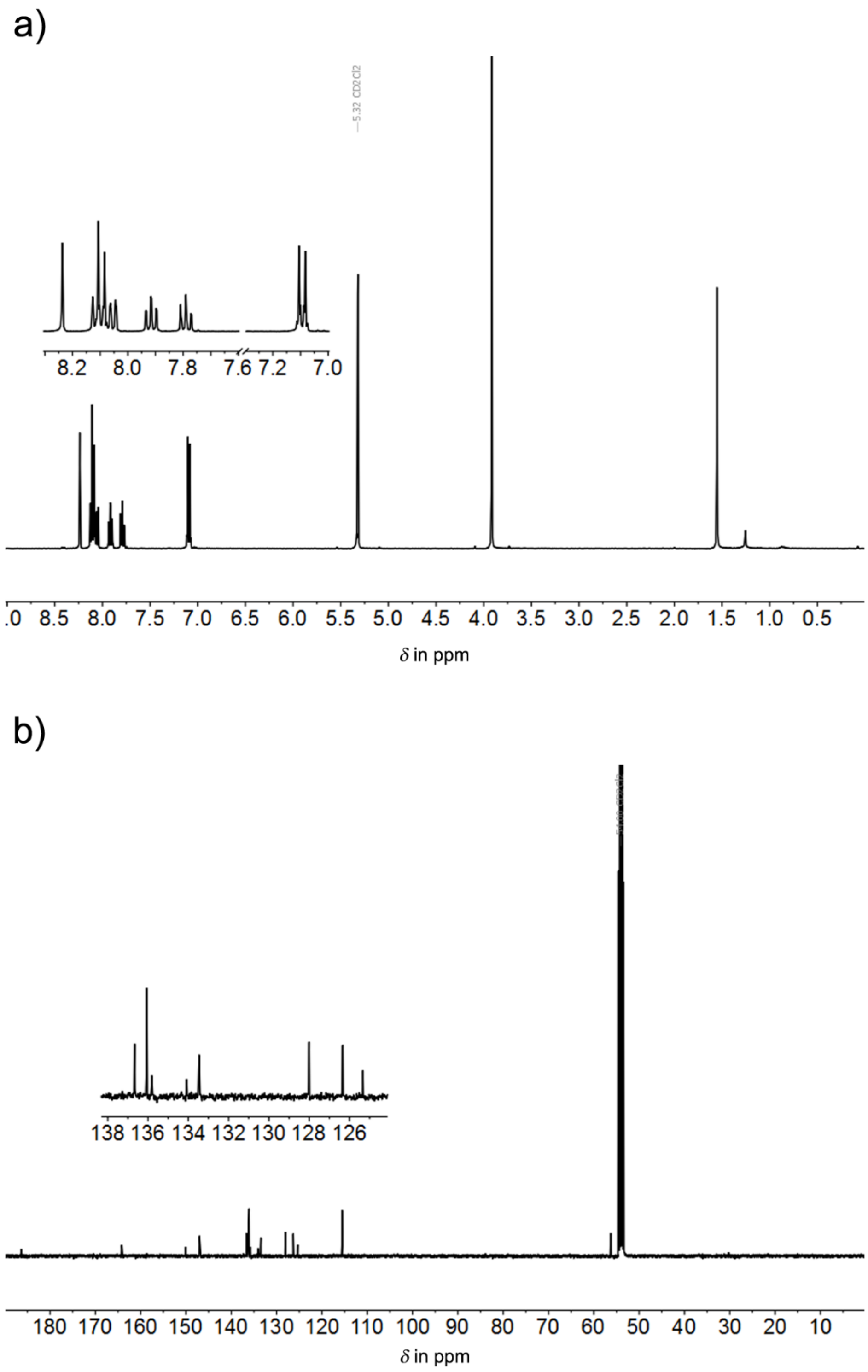

Figure S18 NMR spectra of HTI-SO **2** in CD_2Cl_2 at 23 °C. a) ^{1}H NMR spectrum (400 MHz). b) ^{13}C NMR spectrum (150 MHz).

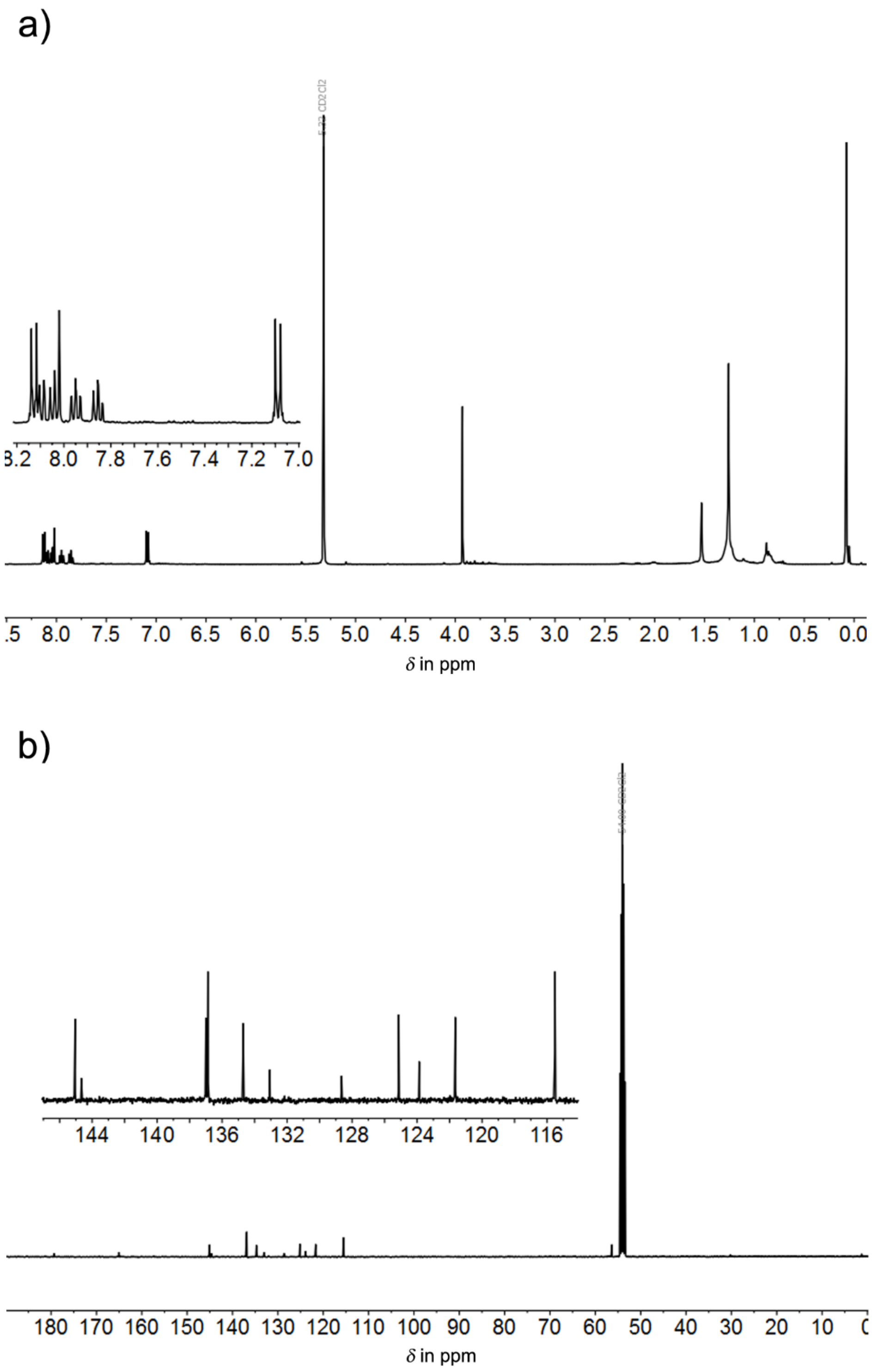

Figure S19 NMR spectra of HTI-SO_2 **2** in CD_2Cl_2 at 23 °C. a) ^{1}H NMR spectrum (600 MHz). b) ^{13}C NMR spectrum (101 MHz).

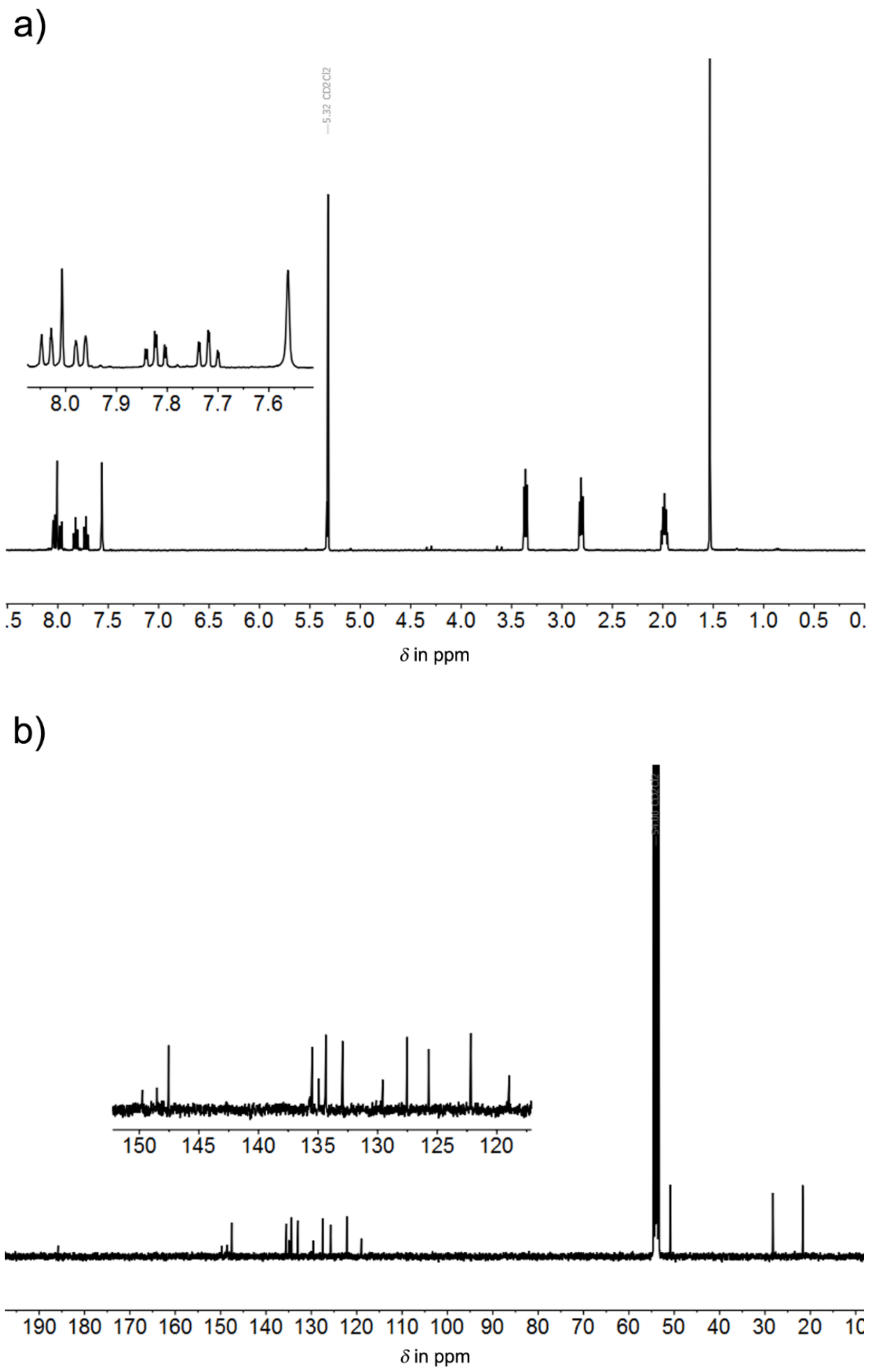

Figure S20 NMR spectra of HTI-SO **3** in CD_2Cl_2 at 23 °C. a) ^{1}H NMR spectrum (400 MHz). b) ^{13}C NMR spectrum (101 MHz).

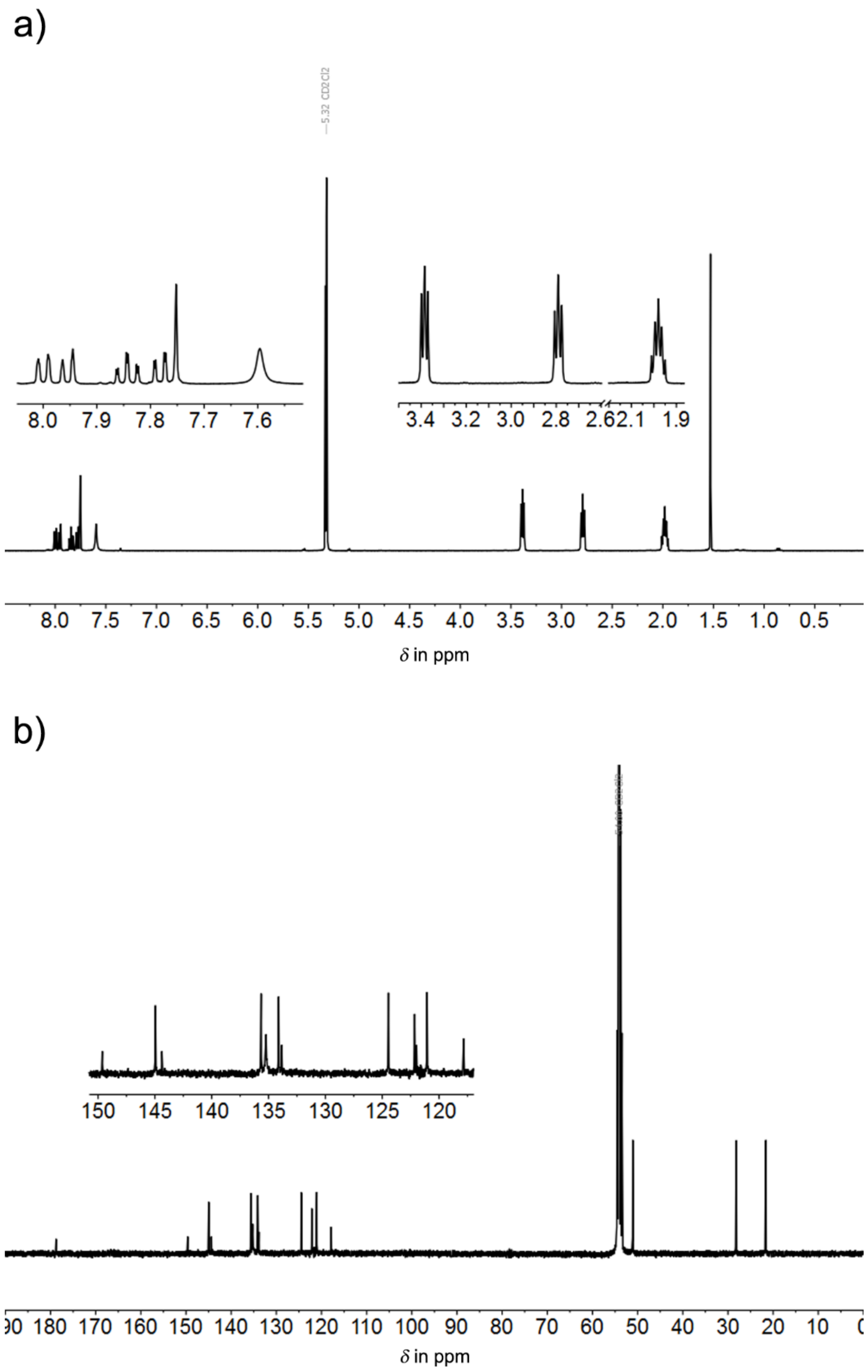

Figure S21 NMR spectra of HTI-SO_2 **3** in CD_2Cl_2 at 23 °C. a) ^{1}H NMR spectrum (600 MHz). b) ^{13}C NMR spectrum (150 MHz).

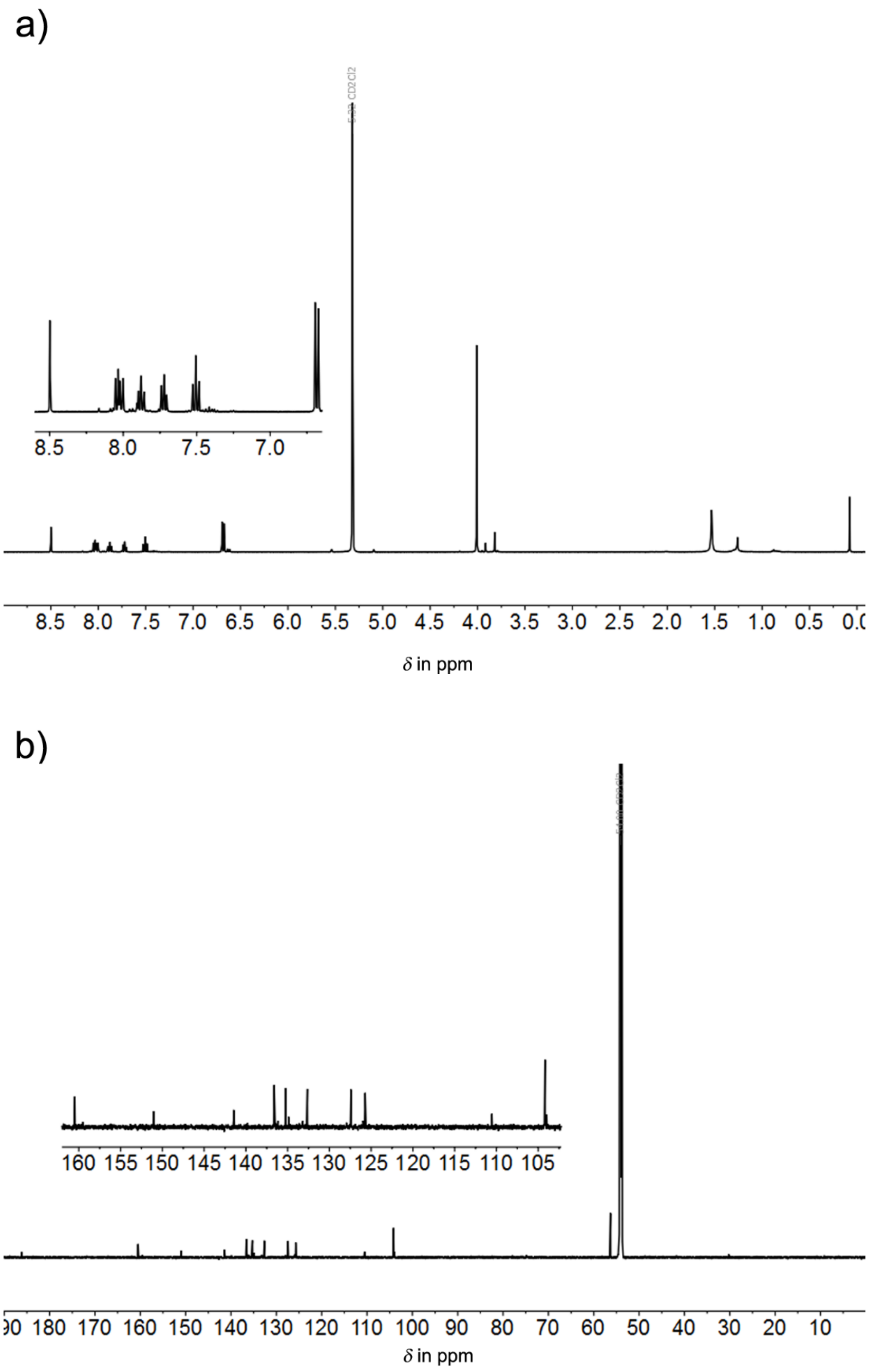

Figure S22 NMR spectra of HTI-SO **4** in CD_2Cl_2 at 23 °C. a) ^1H NMR spectrum (600 MHz). b) ^{13}C NMR spectrum (101 MHz).

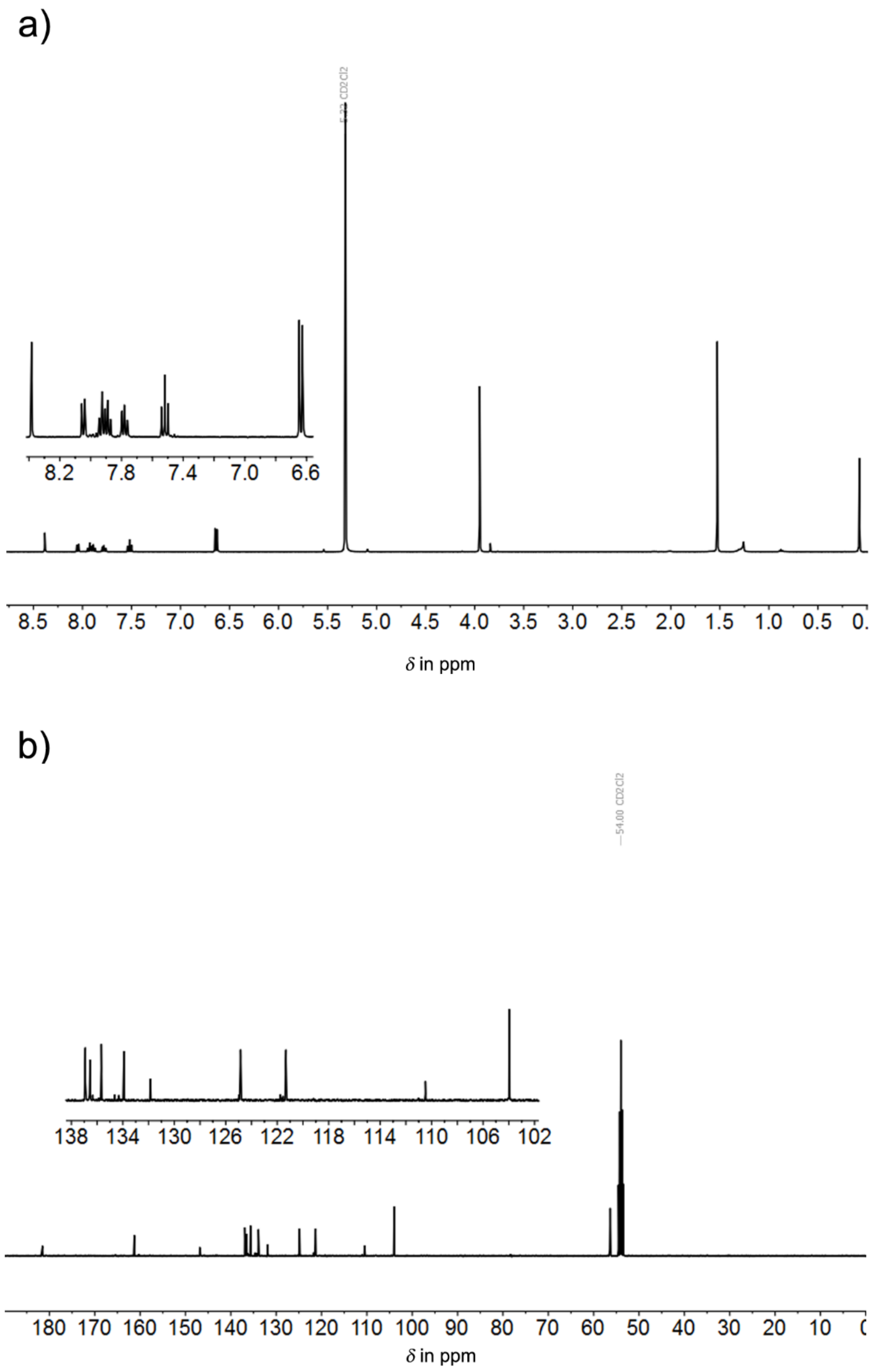

Figure S23 NMR spectra of HTI-SO_2 **4** in CD_2Cl_2 at 23 °C. a) ^{1}H NMR spectrum (400 MHz). b) ^{13}C NMR spectrum (101 MHz).

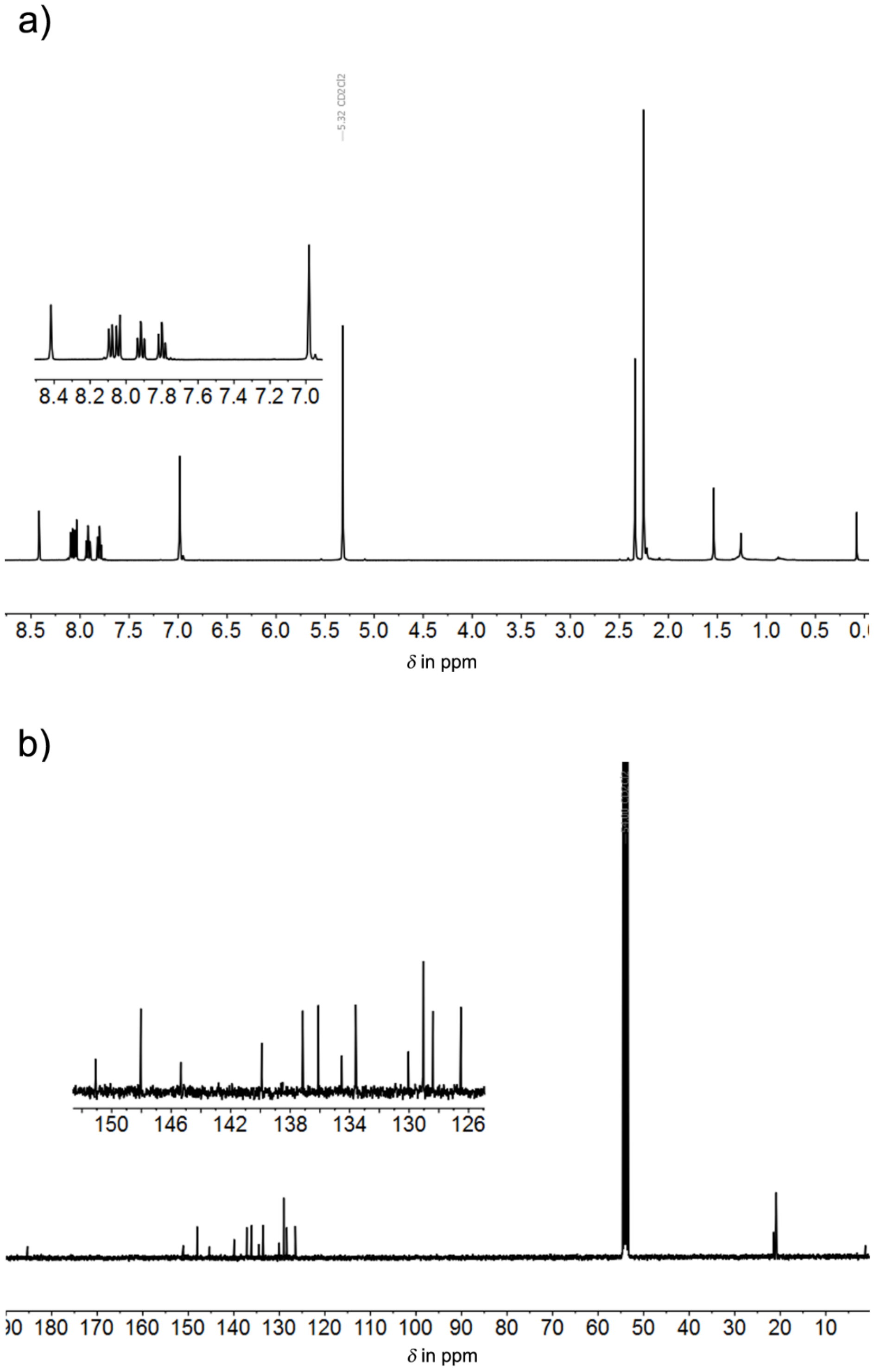

Figure S24 NMR spectra of HTI-SO **5** in CD_2Cl_2 at 23 °C. a) ^1H NMR spectrum (400 MHz). b) ^{13}C NMR spectrum (101 MHz).

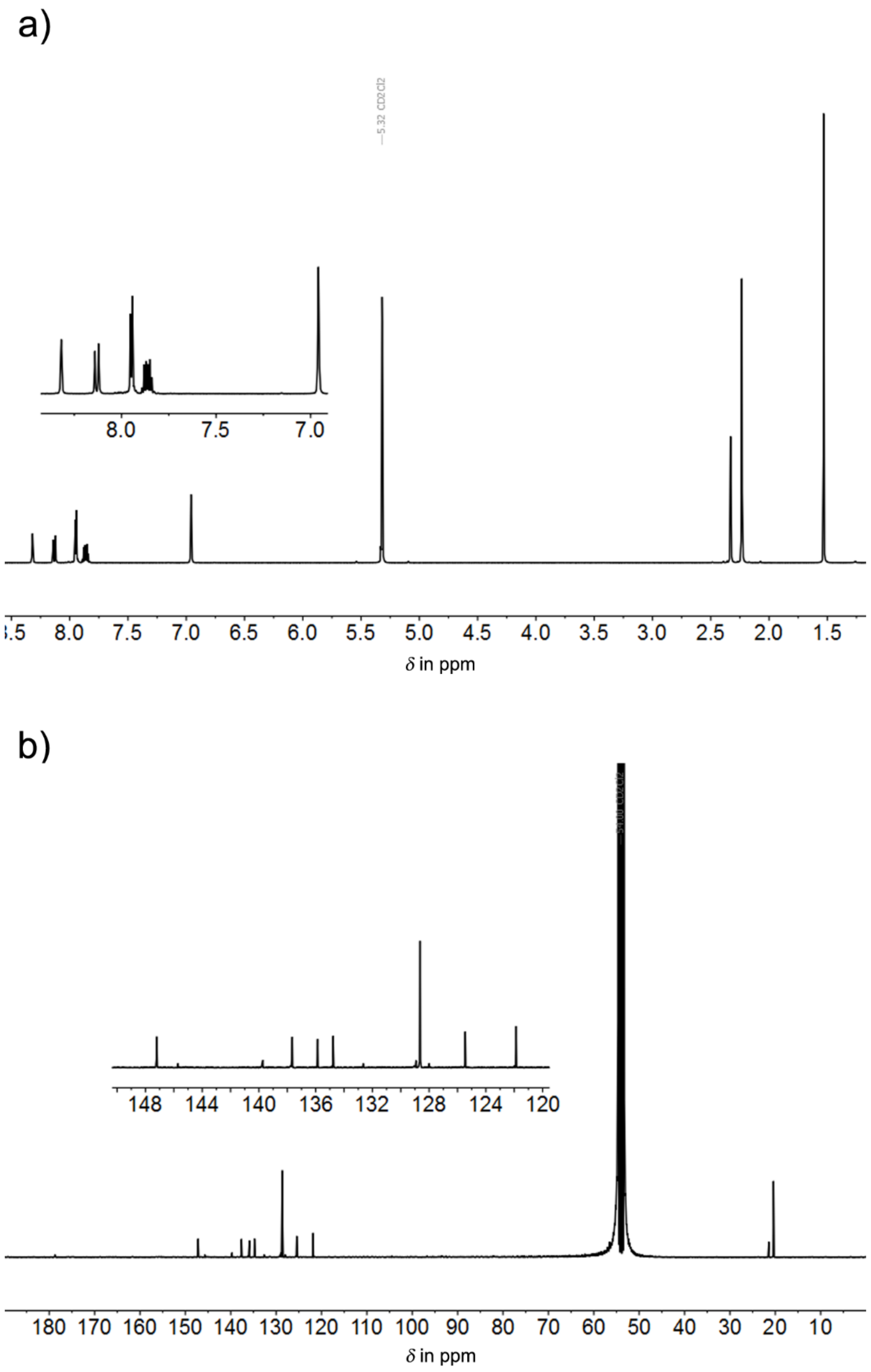

Figure S25 NMR spectra of HTI-SO_2 **5** in CD_2Cl_2 at 23 °C. a) ^{1}H NMR spectrum (400 MHz). b) ^{13}C NMR spectrum (101 MHz).

Crystal Structural Data

X-ray structure determinations: experimental details

The X-ray intensity data of HTI-SO **1**, HTI-SO_2 **1**, HTI-SO **2**, HTI-SO **3**, HTI-SO_2 **3**, HTI-SO **4**, HTI-SO_2 **4** and HTI-SO_2 **5** were measured on a Bruker D8 Venture TXS system equipped with a multilayer mirror optics monochromator and a Mo Kα rotating-anode X-ray tube (λ = 0.71073 Å). Those of HTI-SO_2 **2** and parent HTI **4** were measured on a Bruker D8 Quest IμS system equipped with a multilayer mirror optics monochromator and a Mo Kα micro source X-ray tube (λ = 0.71073 Å). The frames were integrated with the Bruker SAINT software package.[6] Data were corrected for absorption effects using the Multi-Scan method (SADABS).[7] The structures of HTI-SO **1**, HTI-SO_2 **1**, HTI-SO **2**, HTI-SO **3** and HTI-SO_2 **3** were solved and refined using the Bruker SHELXTL Software Package.[8] The structures of HTI-SO_2 **2**, parent HTI **4**, HTI-SO **4**, HTI-SO_2 **4** and HTI-SO_2 **5** were solved with SIR97[9] and refined with SHELXL.[10] All hydrogen atoms have been calculated in ideal geometry riding on their parent atoms. The structure of HTI-SO **2** has been refined as inversion twin. Its volume ratio of twin components refined to 0.85/0.15. The data have been deposited with the Cambridge Crystallographic Data Centre (CCDC) and can be obtained free of charge from: https://www.ccdc.cam.ac.uk/structures/. The CCDC deposition numbers are: 1992231 (HTI-SO **1**), 1992232 (HTI-SO_2 **1**), 1992233 (HTI-SO **2**), 1992234 (HTI-SO_2 **2**), 1992235 (HTI-SO **3**), 1992236 (HTI-SO_2 **3**), 1992237 (parent HTI **4**), 1992238 (HTI-SO **4**), 1992239 (HTI-SO_2 **4**) and 1992240 (HTI-SO_2 **5**).

Table S4 Crystal structural data of parent HTI **4** and HTI-SO **1**.

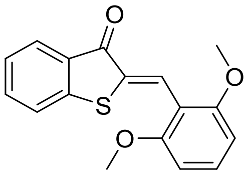

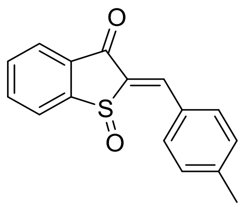

Compound	Parent HTI 4 (CCDC 1992237)	HTI-SO 1 (CCDC 1992231)
net formula	$C_{17}H_{14}O_3S$	$C_{16}H_{12}O_2S$
M_r/g mol^{-1}	298.357	268.32
crystal size/mm	0.263 × 0.138 × 0.030	0.100 × 0.030 × 0.020
T/K	200(2)	101.(2)
radiation	'Mo Kα	MoKα
diffractometer	'Bruker D8Quest'	'Bruker D8 Venture TXS'
crystal system	monoclinic	monoclinic
space group	$P2_1/c$	'P 1 21/c 1'
a/Å	10.9395(4)	4.1048(2)
b/Å	10.8530(4)	21.7155(12)
c/Å	12.2944(5)	13.8850(10)
α/°	90	90
β/°	93.527(2)	95.091(2)
γ/°	90	90
V/Å3	1456.90(10)	1232.80(13)
Z	4	4
calc. density/g cm^{-3}	1.36026(9)	1.446
μ/mm^{-1}	0.229	0.256
absorption correction	multi-scan	Multi-Scan
transmission factor range	0.8620–0.7778	0.91–0.99
refls. measured	16832	21220
R_{int}	0.0507	0.0366
mean σ(I)/I	0.0310	0.0197
θ range	2.51–25.40	3.092–26.363
observed refls.	2029	2179
x, y (weighting scheme)	0.0557, 1.2352	0.0232, 1.0210
hydrogen refinement	constr	constr
refls in refinement	2673	2499
parameters	192	173
restraints	0	0
$R(F_{obs})$	0.0505	0.0334
$R_w(F^2)$	0.1325	0.0832
S	1.059	1.128
shift/error$_{max}$	0.001	0.001
max electron density/e Å^{-3}	0.608	0.337
min electron density/e Å^{-3}	−0.370	−0.382

Table S5 Crystal structural data of HTI-SO **2** and HTI-SO **3**.

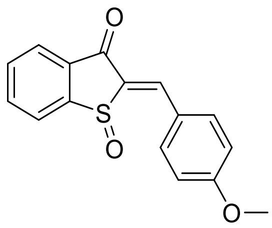

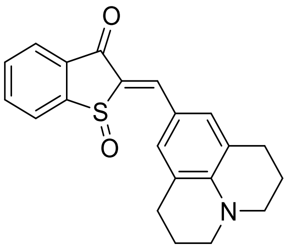

Compound	HTI-SO 2 (CCDC 1992233)	HTI-SO 3 (CCDC 1992235)
net formula	$C_{16}H_{12}O_3S$	$C_{21}H_{19}NO_2S$
M_r/g mol^{-1}	284.32	349.43
crystal size/mm	0.100 × 0.020 × 0.020	0.090 × 0.060 × 0.030
T/K	102.(2)	102.(2)
radiation	MoKα	MoKα
diffractometer	'Bruker D8 Venture TXS'	'Bruker D8 Venture TXS'
crystal system	orthorhombic	triclinic
space group	'P n a 21'	'P -1'
a/Å	12.7484(9)	8.3915(2)
b/Å	24.7986(16)	9.7588(3)
c/Å	4.0111(3)	11.5578(3)
α/°	90	71.4110(10)
β/°	90	69.7240(10)
γ/°	90	68.9410(10)
V/Å3	1268.08(15)	807.88(4)
Z	4	2
calc. density/g cm^{-3}	1.489	1.436
μ/mm^{-1}	0.259	0.215
absorption correction	Multi-Scan	Multi-Scan
transmission factor range	0.87–0.99	0.96–0.99
refls. measured	20607	14224
R_{int}	0.0510	0.0337
mean σ(*I*)/*I*	0.0329	0.0325
θ range	2.937–26.723	2.695–27.479
observed refls.	2538	3084
x, *y* (weighting scheme)	0.0327, 0.6215	0.0370, 0.5672
hydrogen refinement	constr.	constr
refls in refinement	2640	3684
parameters	183	226
restraints	1	0
$R(F_{obs})$	0.0318	0.0378
$R_w(F^2)$	0.0788	0.1014
S	1.097	1.098
shift/error$_{max}$	0.001	0.001
max electron density/e Å^{-3}	0.225	0.405
min electron density/e Å^{-3}	−0.189	−0.368

Table S6 Crystal structural data of HTI-SO **4** and HTI-SO_2 **1**.

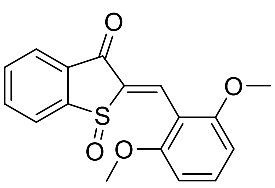

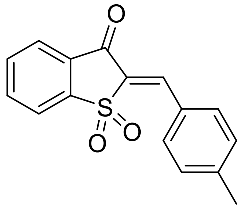

Compound	HTI-SO 4 (CCDC 1992238)	HTI-SO_2 1 (CCDC 1992232)
net formula	$C_{17}H_{14}O_4S$	$C_{16}H_{12}O_3S$
M_r/g mol^{-1}	314.357	284.32
crystal size/mm	0.185 × 0.116 × 0.038	0.080 × 0.060 × 0.040
T/K	123(2)	102.(2)
radiation	Mo Kα	MoKα
diffractometer	Bruker D8Venture	'Bruker D8 Venture TXS'
crystal system	triclinic	monoclinic
space group	*P*1bar	'P 1 21/n 1'
a/Å	6.5610(5)	8.3904(3)
b/Å	8.5089(6)	11.8779(4)
c/Å	13.2424(10)	13.1493(5)
α/°	104.088(2)	90
β/°	95.288(2)	93.5600(10)
γ/°	94.790(2)	90
V/Å3	709.69(9)	1307.93(8)
Z	2	4
calc. density/g cm^{-3}	1.47109(19)	1.444
μ/mm^{-1}	0.244	0.251
absorption correction	multi-scan	Multi-Scan
transmission factor range	0.9300–0.9705	0.95–0.99
refls. measured	12200	22522
R_{int}	0.0353	0.0448
mean σ(*I*)/*I*	0.0276	0.0263
θ range	3.19–25.42	2.966–27.479
observed refls.	2186	2380
x, *y* (weighting scheme)	0.0379, 0.6046	0.0336, 1.1924
hydrogen refinement	constr	constr
refls in refinement	2560	2980
parameters	201	182
restraints	0	0
$R(F_{obs})$	0.0382	0.0390
$R_w(F^2)$	0.0925	0.1042
S	1.044	1.098
shift/error$_{max}$	0.001	0.001
max electron density/e Å^{-3}	0.371	0.326
min electron density/e Å^{-3}	−0.256	−0.434

Table S7 Crystal structural data of HTI-SO_2 **2** and HTI-SO_2 **3**.

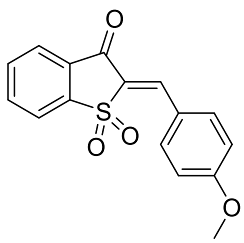

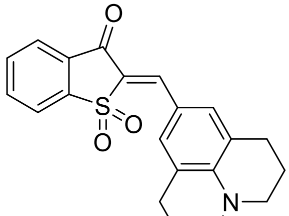

Compound	HTI-SO_2 2 (CCDC 1992234)	HTI-SO_2 3 (CCDC 1992236)
net formula	$C_{16}H_{12}O_4S$	$C_{21}H_{19}NO_3S$
M_r/g mol^{-1}	300.330	365.43
crystal size/mm	0.325 × 0.194 × 0.143	0.090 × 0.060 × 0.030
T/K	173(2)	103.(2)
radiation	Mo Kα	MoKα
diffractometer	Bruker D8Quest	'Bruker D8 Venture TXS'
crystal system	monoclinic	monoclinic
space group	$P2_1/n$	'P 1 21/n 1'
a/Å	7.6940(4)	8.0231(4)
b/Å	12.9912(6)	26.0486(12)
c/Å	13.9403(7)	8.6265(4)
α/°	90	90
β/°	98.5260(17)	104.580(2)
γ/°	90	90
V/Å3	1377.99(12)	1744.80(14)
Z	4	4
calc. density/g cm^{-3}	1.44767(13)	1.391
μ/mm^{-1}	0.248	0.207
absorption correction	multi-scan	Multi-Scan
transmission factor range	0.9291–0.9705	0.95–0.99
refls. measured	28333	33055
R_{int}	0.0492	0.0409
mean σ(*I*)/*I*	0.0272	0.0260
θ range	2.86–27.49	3.198–30.029
observed refls.	2531	4482
x, *y* (weighting scheme)	0.0452, 0.5946	0.0513, 0.7715
hydrogen refinement	constr	constr
refls in refinement	3149	5096
parameters	191	235
restraints	0	0
$R(F_{obs})$	0.0373	0.0364
$R_w(F^2)$	0.0949	0.1046
S	1.062	1.073
shift/error$_{max}$	0.001	0.001
max electron density/e Å^{-3}	0.274	0.447
min electron density/e Å^{-3}	−0.451	−0.417

Table S8 Crystal structural data of HTI-SO_2 **4** and HTI-SO_2 **5**.

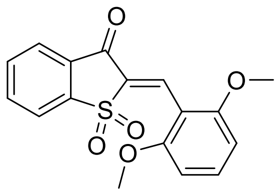

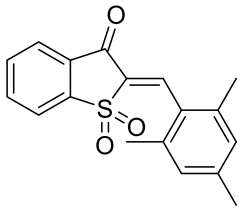

Compound	HTI-SO_2 4 (CCDC 1992239)	HTI-SO_2 5 (CCDC 1992240)
net formula	$C_{17}H_{14}O_5S$	$C_{18}H_{16}O_3S$
M_r/g mol^{-1}	330.356	312.384
crystal size/mm	0.140 × 0.079 × 0.036	0.176 × 0.134 × 0.066
T/K	123(2)	100(2)
radiation	Mo Kα	Mo Kα
diffractometer	Bruker D8Venture	Bruker D8Venture
crystal system	monoclinic	orthorhombic
space group	$P2_1/c$	$Pna2_1$
a/Å	15.1075(13)	16.0862(13)
b/Å	13.1110(11)	11.1660(8)
c/Å	7.4816(6)	8.2665(7)
α/°	90	90
β/°	91.813(2)	90
γ/°	90	90
V/Å3	1481.2(2)	1484.8(2)
Z	4	4
calc. density/g cm^{-3}	1.4814(2)	1.39745(19)
μ/mm^{-1}	0.243	0.228
absorption correction	multi-scan	multi-scan
transmission factor range	0.9245–0.9705	0.6941–0.7455
refls. measured	25587	50179
R_{int}	0.0764	0.0470
mean σ(*I*)/*I*	0.0410	0.0204
θ range	3.11–25.39	3.07–27.13
observed refls.	2052	3091
x, *y* (weighting scheme)	0.0481, 1.6931	0.0409, 0.2717
hydrogen refinement	constr	constr
refls in refinement	2695	3283
parameters	210	202
restraints	0	1
$R(F_{obs})$	0.0491	0.0260
$R_w(F^2)$	0.1207	0.0663
S	1.080	1.063
shift/error$_{max}$	0.001	0.001
max electron density/e Å^{-3}	0.433	0.286
min electron density/e Å^{-3}	−0.434	−0.189

References

[1] B. Maerz, S. Wiedbrauk, S. Oesterling, E. Samoylova, A. Nenov, P. Mayer, R. de Vivie-Riedle, W. Zinth, H. Dube, *Chem. Eur. J.* **2014**, *20*, 13984–13992.

[2] M. Guentner, M. Schildhauer, S. Thumser, P. Mayer, D. Stephenson, P. J. Mayer, H. Dube, *Nat. Commun.* **2015**, *6*, 1–8.

[3] A. Gerwien, M. Schildhauer, S. Thumser, P. Mayer, H. Dube, *Nat. Commun.* **2018**, *9*, 1–9.

[4] S. Wiedbrauk, B. Maerz, E. Samoylova, P. Mayer, W. Zinth, H. Dube, *J. Phys. Chem. Lett.* **2017**, *8*, 1585–1592.

[5] C. Petermayer, S. Thumser, F. Kink, P. Mayer, H. Dube, *J. Am. Chem. Soc.* **2017**, *139*, 15060–15067.

[6] Bruker (2012). *SAINT*. Bruker AXS Inc., Madison, Wisconsin, USA.

[7] Sheldrick, G. M. (1996). *SADABS.* University of Göttingen, Germany.

[8] Sheldrick, G. M. (2015). *Acta Cryst.* A**71**, 3-8.

[9] Altomare, A., Burla, M. C., Camalli, M., Cascarano, G. L., Giacovazzo, C., Guagliardi, A., Moliterni, A. G. G., Polidori, G. & Spagna, R. (1999). *J. Appl. Cryst.* **32**, 115-119.

[10] Sheldrick, G. M. (2015). *Acta Cryst.* C**71**, 3-8.
